# Impact of Milk Fat Globule Membrane and Its Associated Polar Lipids on Markers of Cardiometabolic Disease and Cognitive Function: A Systematic Review and Meta-Analysis of Randomized Controlled Trials in Adults

**DOI:** 10.1016/j.advnut.2026.100694

**Published:** 2026-07-25

**Authors:** Aishwarya S Borkar, Yvanna Todorova, Oonagh Markey

**Affiliations:** School of Sport, Exercise and Health Sciences, Loughborough University, Loughborough, United Kingdom

**Keywords:** cardiometabolic diseases, cognitive function, fasting lipid profile, GRADE approach, lipids, milk fat globule membrane, milk polar lipids

## Abstract

Milk fat globule membrane (MFGM) and its associated milk polar lipids (MPLs) are bioactive components of dairy fat with emerging relevance for cardiometabolic and cognitive health. This systematic review and meta-analysis evaluated the effects of bovine-derived MFGM/MPL on fasting and postprandial cardiometabolic disease risk markers and cognitive function in disease-free adults, compared with isoenergetic dairy products or supplements low in these components. Searches of PubMed (Medical Literature Analysis and Retrieval System Online), Embase, and Cochrane Central identified randomized controlled trials (RCTs) comparing MFGM/MPL-containing foods or supplements with isoenergetic dairy comparators low in these components. Risk of bias (RoB) was assessed using the Cochrane RoB 2.0 tool. Random-effects meta-analyses were performed for outcomes reported in ≥3 studies, and the certainty of evidence for critical outcomes was evaluated using the Grading of Recommendations, Assessment, Development, and Evaluations approach. Of 730 records screened, 13 studies (14 articles) met inclusion criteria (RoB: low to some concerns). Pooled analyses demonstrated that 2 to 16 wk of MFGM/MPL intake (19.8–5000 mg/d) reduced fasting total cholesterol [weighted mean difference (WMD): −0.18 mmol/L; 95% confidence interval (CI): −0.30, −0.07], low-density lipoprotein cholesterol (WMD: −0.12 mmol/L; 95% CI: −0.22, −0.03), apolipoprotein B concentrations (WMD: −0.05 g/L; 95% CI: −0.09, −0.01), and the total cholesterol:high-density lipoprotein (HDL) cholesterol ratio (WMD: −0.17; 95% CI: −0.33, −0.01), with low heterogeneity (*I*^2^ = 0%–9%) and moderate certainty of evidence. No clear effects were observed for fasting circulating HDL cholesterol, triacylglycerol, glucose, insulin, or blood pressure (low certainty). Evidence for emerging fasting cardiometabolic biomarkers, postprandial responses, and cognitive outcomes was limited and synthesized narratively. This review offers, to our knowledge, the most comprehensive and methodologically robust evidence to date that bovine-derived MFGM/MPL modestly improves atherogenic markers in adults, with implications for dietary guidance and future research. Limited data for novel biomarkers and cognitive outcomes highlight priorities for longer-term, adequately powered RCTs to clarify their broader health relevance.

This systematic review protocol was registered at PROSPERO as CRD42025636269.

**Statement of significance**

This systematic review provides, to our knowledge, the most comprehensive synthesis and meta-analysis to date evaluating whether bovine-derived milk fat globule membrane or membrane-associated milk polar lipids influence markers of cardiometabolic disease risk and cognitive function among disease-free adults, across both fasted and postprandial states, compared with dairy products or supplements low in these components. By integrating subgroup analyses across baseline health status, intervention duration, and dose, and applying the Grading of Recommendations, Assessment, Development, and Evaluations approach to assess the certainty of evidence for critical outcomes, this review delivers a rigorous and policy-relevant synthesis to inform nutrition guideline development, dietary guidance, and future research priorities.

## Introduction

Cardiometabolic diseases (CMDs), including cardiovascular diseases (CVDs), remain leading causes of mortality worldwide [1]. Diet is a key modifiable risk factor, and circulating lipid markers are primary targets of many dietary interventions [2,3]. Full-fat dairy has traditionally been regarded as adverse for cardiometabolic health because of its high content of long-chain SFAs, and their established cholesterol-raising effects [4,5]. Consequently, most dietary guidelines continue to recommend replacing full-fat dairy products with low-fat or fat-free alternatives to limit SFA intake [6,7]. However, accumulating evidence from large prospective cohort studies and systematic reviews with meta-analyses challenges this traditional nutrient-centric paradigm, indicating that higher intakes of full-fat dairy foods (with the exception of butter) are not associated with increased CVD risk [[8], [9], [10], [11]]. Physiologic responses to dietary SFAs appear to depend strongly on the dairy food matrix rather than SFA content alone [12]. For example, a systematic review and meta-analysis (SRMA) of randomized controlled trials (RCTs) demonstrated that cheese produces more favorable effects on total and LDL cholesterol than butter when isoenergetically substituted, highlighting the influence of matrix-related structural components [13]. One such component is the trilayered milk fat globule membrane (MFGM), which encapsulates milk fat globules and is rich in bioactive milk polar lipids (MPLs) including glycerophospholipids and sphingolipids and membrane-associated proteins [14].

The MFGM is naturally abundant in dairy products in which milk fat globules remain intact, such as cream [14]. However, processing techniques, particularly churning and homogenization, disrupt the fat globule structure and release a substantial proportion of MFGM components into byproducts such as buttermilk and cheese whey (for review, see [15]). These side streams can then be fractionated to produce MFGM-enriched functional ingredients [16].

Mechanistic evidence suggests that MFGM-derived MPLs, particularly sphingomyelin (SM), are enriched in long-chain saturated fatty acyl groups and interact with cholesterol in the intestinal lumen. These interactions have been shown in experimental models to reduce intestinal absorption and increase fecal cholesterol excretion and may contribute to lower hepatic cholesterol and triacylglycerol (TG) accumulation and to the modulation of postprandial lipid metabolism (for review, see [17]). These biological properties have stimulated interest in the potential of MFGM and MPLs to favorably influence cardiometabolic risk. Beyond traditional lipid markers [[18], [19], [20], [21], [22], [23], [24], [25], [26], [27]], several RCTs have investigated the influence of MFGM on other cardiometabolic biomarkers, including glycemic control [[21], [22], [23], [24],^26^] and blood pressure (BP) [[22], [23], [24],^26^,^28^], although findings remain inconsistent and have not been systematically synthesized. In parallel, MFGM and its constituent MPLs play recognized roles in early life neurocognitive development [29], and emerging RCTs have begun to investigate their potential relevance for maintaining cognitive function or mitigating age-related cognitive decline in adulthood (for detailed review, see [30]).

A 2024 SRMA of 6 RCTs reported that MFGM, delivered alone or, in 1 study, as part of a multicomponent intervention involving a multi-nutrient-fortified milk drink and exercise, significantly reduced fasting total cholesterol (TC) and LDL cholesterol, with no effect on HDL cholesterol or TG concentrations [31]. The present SRMA builds on this evidence by focusing specifically on bovine-derived MFGM administered without co-interventions and by evaluating a broader range of clinically relevant cardiometabolic outcomes. For example, circulating LDL cholesterol is only partly representative of atherogenic lipid burden, whereas apolipoprotein B (apo B) directly reflects the total number of atherogenic lipoprotein particles and is a stronger predictor of CVD risk than LDL cholesterol alone [32,33]. As individuals spend much of the day in the non-fasted state, and elevated postprandial TG concentrations independently predict CMD risk, we also evaluated available evidence on the effects of MFGM/MPL on postprandial metabolism [[34], [35], [36]]. To strengthen the certainty and interpretation of findings, we applied the Grading of Recommendations, Assessment, Development, and Evaluation (GRADE) approach [37] and conducted subgroup analyses to examine whether baseline health status, intervention delivery format, duration, and MFGM/MPL dose modify the observed effects. Thus, this SRMA addresses whether consumption of bovine-derived MFGM or membrane-associated MPLs influences CMD risk markers or cognitive function in adults, in both the fasted and postprandial states, compared with isoenergetic dairy products or supplements low in MFGM/MPL. Unlike the single prior SRMA [31], we integrated lipids, apolipoproteins, and broader fasting and postprandial cardiometabolic markers, alongside gut microbiota and fecal lipid–related metabolites and cognitive function outcomes, within a single, harmonized evidence framework–.

## Methods

### Registration

This systematic review was reported in accordance with PRISMA guidelines [38], and the protocol was prospectively registered with PROSPERO (www.crd.york.ac.uk/PROSPERO); registration number: CRD42025636269.

### Study eligibility criteria

Eligibility criteria of the studies were defined using the population, intervention, comparator, outcome, and study design (PICOS) framework, as presented in Table 1. Full-text, peer-reviewed RCTs in English were eligible, with no restriction on publication date. Studies were required to involve weight-stable adults (≥18 y) who were disease-free, cognitively healthy, or at risk of developing CMDs or dementia (e.g., elevated lipid profile, BMI ≥25 kg/m^2^, and mild cognitive impairment). Eligible RCTs (parallel and crossover) also had to meet the following criteria: *1*) the intervention provided bovine-derived MFGM or MPL either through food-based administration (e.g., dairy products) or supplement-based administration (e.g., whey protein–based concentrate); *2*) the comparator was an isoenergetic alternative dairy product or supplement with low or no MFGM/MPL content; *3*) reported ≥1 marker of CMD risk and/or cognitive function in the fasted or postprandial state. For postprandial studies, eligibility required the following: *1*) a test meal containing 30 to 80 g total fat, including MFGM/MPL, with comparator meals containing similar amounts of fat; and *2*) an assessment of relevant outcomes over a 4 to 8 h postprandial period, consistent with previous methodology [13].

**TABLE 1:** Inclusion and exclusion criteria for the review on the impact of MFGM and associated polar lipids on markers of cardiometabolic disease and cognitive function in the fasted and postprandial state

Parameter	Inclusion	Exclusion
Population or participants	Eligible studies will include human adults aged ≥18 y who are disease-free, cognitively healthy, or at risk of developing cardiometabolic disease (e.g., elevated lipid profile, BMI ≥ 25 kg/m^2^) or dementia (e.g., mild cognitive impairment). No restrictions on sex, ethnicity, or study setting.	Studies including adults with chronic diseases such as CVD, diabetes, liver disease; neurological diseases (e.g., Alzheimer’s and other forms of dementia); severe cognitive impairment, or serious mental illness known to affect cognition (e.g., schizophrenia, schizoaffective disorder, and bipolar disorder). Include those prescribed medication likely to interfere with study outcomes, including those for cholesterol, lipid, inflammation, immune function, blood pressure management, or cognitive enhancement. Non-weight-stable participants (>5 kg weight change in last 6 mo).
Independent variable (intervention)	Diets administered through the provision of foods or supplements, including food-based (e.g., dairy products) or supplement-based (e.g., whey protein–based concentrate) interventions that incorporate MFGM or membrane-associated MPL. For postprandial studies, test meals will be required to contain 30–80 g total fat (including fat from MFGM/MPL) and be matched with comparator meals for total fat content. Relevant outcomes must be assessed over a 4–8 h postprandial period.	Studies with multi-nutrient-fortified products or supplements combining MFGM/MPL with other nutrients [e.g., vitamin D, phytosterols, conjugated linoleic acid (18:2n–6), ω-3 fatty acids, or other micronutrients] or if co-interventions such as drug, diet, or exercise are used.
Comparator	Energy-matched (isoenergetic) quantities of alternative dairy products or supplements that are devoid of, or contain low quantities of, MFGM or MFGM-associated MPL	Studies where energy exchange between dietary arms differs significantly or if the nutrient composition of the study arms cannot be accurately determined.
Dependent variable (outcome)	Cardiometabolic outcomes assessed in the *1*) fasted and *2*) postprandial state: blood lipids/lipoproteins (total, LDL, HDL, VLDL cholesterol, triacylglycerol, apolipoprotein A-I, apolipoprotein B, lipoprotein(a)); markers of insulin resistance and glucose metabolism; inflammatory markers (interleukin-6, C-reactive protein, etc.); blood pressure and vascular function; gut microbiota and metabolites. Cognitive outcomes assessed in the *1*) fasted and *2*) postprandial state: global cognitive function or domain-specific measures (e.g., memory, executive function, and verbal fluency). Additional: circulating biomarkers of cognitive function, e.g., brain-derived neurotropic factor, and amyloid-β.	Studies without relevant outcomes.
Study design	Randomized controlled trials (parallel or crossover), including chronic and acute/postprandial designs.	Cross-sectional studies, case-control studies, pre–post studies without a control, narrative reviews, systematic reviews, meta-analyses, and acute-within-chronic RCT designs (i.e., studies assessing acute responses during a chronic intervention).
Publication status	Full-text articles in peer-reviewed journals.	Unpublished studies or gray literature such as conference abstracts, reports, letters, and editorials.
Language	English	Articles not in English.

Abbreviations: CVD, cardiovascular disease; MFGM, milk fat globule membrane; MPL, milk polar lipid; RCT, randomized controlled trial.

Exclusion criteria included the following: *1*) studies comparing MFGM/MPL consumption to nondairy food comparators only; *2*) studies using multi-nutrient-fortified products or supplements combining MFGM with other nutrients [e.g., vitamin D, phytosterols, and omega-3 (n–3) fatty acids] or co-interventions (diet, drug, or exercise); and *3*) studies using acute-within-chronic study designs (i.e., studies that assess acute postprandial responses within the context of a longer-term intervention).

### Search strategy

Electronic literature searches were conducted in PubMed (Medical Literature Analysis and Retrieval System Online), Cochrane Central, and Embase in June 2025, with supplementary searches continued until 23 September, 2025. The search strategy was developed by ASB and OM using the PICOS framework and reviewed by an independent academic librarian. It was refined through scoping searches. Reference lists of relevant review articles and included RCTs were also screened. Citation alerts were set up in Google Scholar to identify eligible studies published before review completion. Full search strategies for each database are presented in Supplemental Table 1.

### Study selection

Records were imported into Covidence software (Veritas Health Innovation Ltd), and duplicates were removed before screening. Using a multiple-pass approach, titles and abstracts were screened independently by 2 reviewers (first pass), followed by full-text screening of potentially eligible articles (second pass) by independent reviewers (ASB, YT, and OM). Discrepancies were resolved through discussion, and reasons for exclusion at full-text screening were documented.

### Data extraction process

Data were extracted into an Excel form, which was piloted by 2 reviewers (ASB and OM) on 10% of the included studies and finalized before use. Extraction was conducted by a single reviewer (ASB) with all records (100%) checked for completeness and accuracy by an independent reviewer (OM). Any discrepancies were resolved through discussion. Extracted information included first author name, publication year, country, study design, location, participant characteristics, intervention and comparator details, relevant outcomes (including assessment method, analytical sample size, details of power calculation, adjustment for confounders, and effect estimates), and funding sources. Details of the extracted variables are provided in Supplemental Table 2. Where available, means, SDs, SEs, or 95% confidence intervals (CIs) were extracted for baseline, post-intervention and change-from-baseline values. For multi-arm RCTs, only data from relevant intervention and comparator arms were extracted. Study protocols, trial registrations, and Supplemental Materials were also reviewed for data extraction if required information was not presented in the included articles.

### Risk-of-bias assessment

Risk of bias (RoB) for each included study was independently assessed at the outcome level by 3 reviewers (ASB, YT, and OM) using the Cochrane RoB 2.0 tool [39]. The 5 domains of bias and the overall RoB were evaluated and classified as low, moderate (some concerns), or high RoB (see Supplemental Table 3 for details**)** [39]. For crossover studies, potential bias arising from period and carryover effects was also assessed. Washout periods of ≥14 d were deemed sufficient regardless of intervention length [13,39]. Full-text articles were the primary source for these assessments; however, Supplemental Materials or trial registrations were consulted where necessary [39]. Study-level and summary RoB figures were created using the Risk-Of-Bias VISualization (robvis) tool [40].

### Data synthesis methods

#### Eligibility and preparation for synthesis

For all included studies, baseline and post-intervention outcomes were extracted and summarized as means and SDs in International System of Units where possible. When information was incomplete (e.g., missing timepoints or outcomes not reported as mean ± SD), necessary values were imputed by converting SEs to SDs, or by approximating means and SDs from medians and IQRs or 95% CIs, following Cochrane Handbook guidance [41]. When numerical values were not reported in the text, data were extracted from available figures using WebPlotDigitizer (version 4.8, Ankit Rohatgi; https://apps.automeris.io/wpd4/). Where required, outcome measures were converted to standard concentration units using established conversion factors. Because no response was obtained after contacting the authors of 1 full-text article [42], BP data from this study were omitted because no numerical values were provided in the publication. Crossover studies were analyzed using the same approach as parallel studies, by comparing intervention with control periods. As individual paired data were not consistently available, this approach does not account for within-participant correlation and therefore introduces a unit-of-analysis error. However, this method is considered conservative and has been used previously in systematic reviews when paired analyses are not feasible [41]. Where trials permitted heterogeneous populations and subgroup analyses were not reported or could not be obtained, eligibility was assessed using the trial’s published inclusion and exclusion criteria together with relevant baseline biochemical characteristics [41].

### Statistical analysis

When exposures could be harmonized for the same outcome, studies were included in meta-analyses. A priori, a minimum of 3 studies reporting the same outcome was required to produce a forest plot. For each outcome, studies were included in the meta-analysis using the effect measure reported (post-intervention values or change from baseline). In line with Cochrane guidance [41], post-intervention and change-from-baseline estimates were pooled together. When both were available, change-from-baseline values were prioritized.

Analyses were conducted using Review Manager (RevMan, version 9.8.2; Cochrane). As all relevant outcomes were continuous, effect sizes were quantified using an inverse-variance random-effects model. Intervention effects are expressed as weighted mean differences (WMDs) and 95% CIs of post-intervention and change-from-baseline outcomes between study arms [41]. The restricted maximum likelihood estimator was applied to estimate heterogeneity variance, and the Hartung–Knapp–Sidik–Jonkman (HKSJ) correction was applied to estimate the 95% CIs of the summary effects [13,43]. Statistical heterogeneity was quantified using the *I*^2^ statistic and interpreted using thresholds of low (25%), moderate (50%), and high (75%) heterogeneity, with values of ≥50% considered substantial [41]. For meta-analyses including ≥10 studies, funnel plots were generated to explore small-study effects and possible publication bias, in line with Cochrane guidance [41]. Funnel plot asymmetry was evaluated using Egger’s regression test [44] in R studio (version 2024.04.0+735; Posit Software, PBC), with a *P* value of <0.05 considered statistically significant.

Subgroup analyses were exploratory and conducted to examine whether effects varied by intervention delivery format, dose, or study characteristics. These analyses were not powered for definitive conclusions and should be interpreted as hypothesis generating. Subgroup analyses were conducted in accordance with the PROSPERO protocol, with categories informed by a priori considerations and refined following evaluation of the included studies before data analysis. Analyses were performed only for outcomes with sufficient data (≥5 studies overall and ≥2 studies per subgroup level) to ensure meaningful interpretation and were not conducted where these criteria were not met. Subgroups were defined as follows: *1*) baseline health status: healthy vs. at risk (based on overweight/obesity or elevated LDL cholesterol concentrations); *2*) intervention delivery format: food-based (native MFGM delivered via whole dairy matrices) vs. hybrid (processed supplementation incorporated into a dairy food) vs. supplement-based (processed MFGM/MPL delivered via powders, tablets, or formulated drinks); *3*) intervention duration: short- (≤4 wk) vs. long-term (>4 wk); and *4*) intervention dosage: low dose (<300 mg/d) vs. high dose (≥300 mg/d). Intervention delivery format analyses were not undertaken because the prespecified data sufficiency criteria were not met.

Formal tests for between-subgroup differences (interaction effects) were conducted where data permitted. Where there were insufficient comparable RCTs to meta-analyze effect estimates, quantitative data were synthesized narratively in line with synthesis without meta-analysis (SWiM) guidelines [45].

### Sensitivity analyses

Sensitivity analyses were conducted to evaluate the robustness of pooled estimates for all lipid outcomes. Studies were flagged for potential baseline imbalance if the mean difference between intervention and control groups exceeded ±10% of the clinically desirable upper limit, based on United Kingdom clinical guidelines. The thresholds applied were TC ± 0.52 mmol/L (upper limit 5.2 mmol/L), LDL cholesterol ± 0.3 mmol/L (upper limit 3.0 mmol/L), HDL cholesterol ± 0.14 mmol/L (target 1.4 mmol/L), TG ± 0.17 mmol/L (upper limit 1.7 mmol/L), and TC:HDL cholesterol ratio ± 0.4 units (target 4.0) [46,47]. Based on these criteria, baseline imbalance was identified for LDL and HDL cholesterol in the studies by Ohlsson et al. [18] and Calvo et al. [24] and for TG in Ohlsson et al. [18], Severins et al. [27], and Calvo et al. [24].

A separate sensitivity analysis excluding the study by Zajac et al. [25] was conducted because of the absence of individual-level reporting of diabetes status and glucose-lowering medication use. According to the trial’s published eligibility criteria, participants were excluded if glycated hemoglobin (HbA1c) was ≥6.5% (≥48 mmol/mol) in individuals without diagnosed diabetes or ≥7.0% (≥53 mmol/mol) in those with diagnosed type 2 diabetes who were medically managed for ≥6 mo. As individual-level subgroup data restricted to participants without diabetes were unavailable despite author contact, eligibility for inclusion in the meta-analysis was assessed using the trial’s published inclusion and exclusion criteria together with objectively reported baseline biochemical characteristics, consistent with Cochrane Handbook guidance [41]. In this trial, mean baseline HbA1c values across study arms ranged from 5.46% to 5.54%, with small SDs (∼0.3% points) and balanced distributions between intervention groups, all within the normoglycemic range (<5.7%). Clinically, such HbA1c values are not consistent with the inclusion of a meaningful proportion of individuals with treated type 2 diabetes, even under conditions of intensive glycemic control, and therefore argue against substantive confounding from glucose-lowering therapy [48]. Nevertheless, the study was excluded in sensitivity analyses to formally assess the robustness of pooled estimates to any residual uncertainty.

### Certainty of evidence

The GRADE approach was used to assess the certainty of evidence for fasting outcomes that were prespecified as critical to public health decision making [49], in line with GRADE guidance [37]. Two reviewers (OM and ASB) independently rated the evidence as high, moderate, low, or very low, followed by discussion and consensus. In accordance with GRADE guidance, certainty ratings were downgraded based on the following domains: RoB, inconsistency, indirectness, imprecision, and other considerations [37]. Evidence profile tables were generated for each outcome using GRADEpro GDT software (GRADEpro Guideline Development Tool; Evidence Prime, Inc.).

## Results

### Study selection and characteristics

**FIGURE 1:**
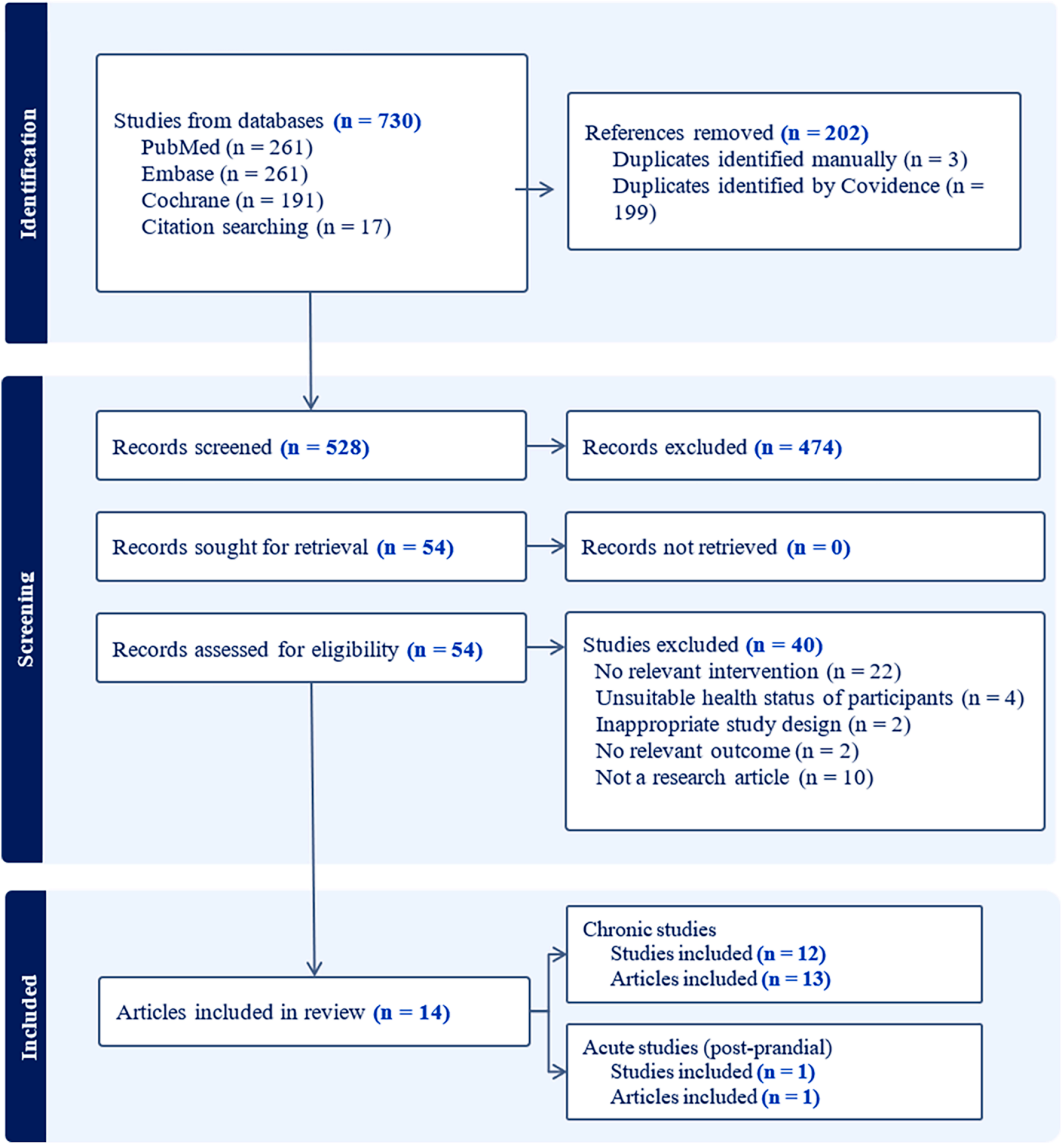
Flowchart of study search and selection for the review of the impact of milk fat globule membrane and its associated milk polar lipids on cardiometabolic disease and cognitive function risk markers in the fasted and postprandial state.

The study search and selection process is summarized in the PRISMA flow diagram (Figure 1). A total of 730 records were identified through database and citation searching. After the removal of 202 duplicates, 528 records remained for screening. After title and abstract screening (phase 1), 474 records were excluded. Full texts of 54 records were assessed for eligibility (phase 2), of which 40 were excluded for the following reasons: no relevant intervention (*n* = 22), unsuitable health status of participants (*n* = 4), inappropriate study design (*n* = 2), no relevant outcome (*n* = 2), and not a research article (*n* = 10).

The characteristics of the included studies are presented in Table 2. In total, 13 studies (reported across 14 articles) met the inclusion criteria. Of these, 12 [[18], [19], [20], [21], [22], [23], [24], [25], [26], [27], [28],^42^,^50^] reported cardiometabolic outcomes, whereas 4 studies [24,25,42,51] assessed outcomes related to cognitive function. Two articles [20,28] reported outcomes from the same trial and were therefore treated as a single study for data synthesis and RoB assessment. Of the included studies, 12 chronic (fasting) studies were eligible for meta-analyses, whereas 1 acute (postprandial) study was summarized narratively. Several fasting and postprandial studies appeared to meet the inclusion criteria but were excluded for predefined reasons [22,23,27,[52], [53], [54], [55], [56], [57], [58]]. Full details are provided in the Supplemental Results.

**TABLE 2:** Synthesis of characteristics of eligible RCTs on the impact of milk fat globule membrane and its associated polar lipids on cardiometabolic disease and cognitive function risk markers in the fasted state^1^

Covidence study ID	First author, y (country)	Health status	Study completers, *n* (%, M/F)	Age (mean or range)	BMI (mean or range) (kg/m^2^)	Study design, type of dietary intervention, duration of intervention, and washout	Study arms, *n*	Intervention	Comparator	Relevant outcomes	Industrial funding (yes/no)	Overall RoB
Ohlsson 2009	Ohlsson et al. [18], 2009 (Sweden)	Healthy	MPL-enriched buttermilk: 29 (69/31) Control: 19 (68/32)	20–65 y	MPL-enriched buttermilk: 24.1 Control: 24.7	Parallel, semi-controlled, intervention: 4-wk, (1-wk run-in)	2	350 mL buttermilk drink enriched with Lacprodan PL-20 (1950 mg SL/d) Supplied by: Arla Foods Amba	Placebo drink made with skim milk powder with PL replaced by 2/3 egg PL isolate + 1/3 butter oil. (238 mg SL/d)	TC, LDL-C, HDL-C, TG, Apo A-I, Apo B, Apo B:Apo A-I, Lp(a), PO: NR	Yes	Some concerns
Schubert 2011	Schubert et al. [50], 2011 (Germany)	Healthy, chronically stressed	75 (100/0)	30–51 y	1% MPL drink: 26.46 0.5% MPL drink: 28.53 Control drink: 26.90	Parallel, placebo-controlled, semi-controlled, intervention: 12 wk	3	250 mL/d bovine milk drink enriched with 0.5% and 1% MPL (Lacprodan PL-20), (150 mg and 300 mg MPL/d, respectively) Lacprodan PL-20 supplied by: Arla Foods Ingredients, Viby, Denmark	250 mL/d placebo bovine milk drink	Memory performance, PO: NR	Yes	Some concerns
Conway 2013^2^	Conway et al. [20], 2013 (Canada)	Healthy LDL-C <5 mmol/L BP <140/90 mmHg 10 y Framingham risk <10%	34 (44/56)	18–65 y	≤35	Crossover, placebo-controlled, semi-controlled, intervention: 4-wk, (no washout)	2	45 g/d buttermilk powder mixed with 250 mL water (187.5 mg MPL/d) Supplied by: Westland Milk Products, Hokitika, New Zealand	45 g/d macro/micronutrient-matched dairy-based powder (lacking MFGM), mixed with 250 mL water (34.6 mg PL/d)	TC, PO: LDL-C, HDL-C, TG, TC:HDL-C, Apo B100, Apo B48, PCSK9, CRP	Yes	Some concerns
	Conway et al. [28], 2014 (Canada)									SBP DBP		
Ramprasath 2013	Ramprasath et al. [19], 2013 (Canada)	Healthy Plasma TC 5.2 mmol/L; LDL-C: 3.1 mmol/L	10 (50/50)	18–45 y	23 ± 2	Crossover, fully controlled, intervention: 2-wk (4-wk washout)	2	Diet with 1 g milk SM, dissolved in corn oil and added to the diet (1000 mg SM/d) SM supplied by: Avanti Polar Lipids, Inc., Alabaster, Alabama, United States	Identical diet without SM	TC, LDL-C, VLDL-C, HDL-C, TG, TC:HDL-C, PO: NR	No	Some concerns
Severins 2014	Severins et al. [27], 2014 (Netherlands)	Mildly hypercholesterolemic Elevated serum TC: 5.3–8.6 mmol/L	Buttermilk: 23 (57/43) Skimmed milk: 25 (44/56)	18–70 y	23–34	Parallel, placebo-controlled, semi-controlled, intervention: 12-wk (2-wk run-in)	4 Extracted: 2	80 mL/d traditional buttermilk (0.09% MPL) Supplied by: Coppelmans BV, Waalre, Netherlands	80 mL/d skimmed milk (0.01% PL)	TC, PO: LDL-C, HDL-C, TG, TC:HDL-C, Apo A-I, Apo B100, CRP	Yes	Low risk
Rosqvist 2015	Rosqvist et al. [21], 2015 (Sweden)	OW/OB, Elevated plasma LDL-C: 2.0–4.5 mmol/L	MFGM diet: 26 (31/69) Control diet: 20 (20/80)	20–70 y	25–37	Parallel, semi-controlled, intervention: 8-wk	2	1 dL/d whipping cream (rich in intact MFGM, 19.8 mg MPL/d) + 1 dL/d fat-free milk + 1 scone Whipping cream supplied by: Skånemejerier AB, Sweden	1 dL/d fat-free milk (1.3 mg PL/d) + 1 scone/d	TC, PO: LDL-C, VLDL-C, HDL-C, non-HDL-C, TG, TC:HDL-C, LDL:HDL, Apo A-I, Apo B, Apo B:Apo A-I, PCSK9, CRP, glucose, insulin, HOMA-IR	Yes	Some concerns
Hari 2015	Hari et al. [26], 2015 (Japan)	Healthy	MFGM: 16 (50/50) Placebo: 16 (50/50)	20–64 y	MFGM: 23.1 ± 1.7 Placebo: 23.0 ± 3.3	Parallel, placebo-controlled, semi-controlled, intervention: 4-wk	2	6.5 g MFGM/d (231 mg SM/d) in the form of a tablet Supplied by: NR	6.5 g/d whole milk powder in the form of a tablet (4 mg SM/d)	TC, LDL-C, HDL-C, TG, glucose, NEFA, SBP, DBP, PO: NA	NR	Low risk
Weiland 2016	Weiland et al. [22], 2016 (Germany) Trial 1	OW/OB, fasting glucose < 7.0 mmol/L	62 (100/0)	50–76 y	≥ 27	Parallel, placebo-controlled, semi-controlled, intervention: 8-wk	2	200 mL/d semi-skimmed milk (1.5% fat) + 2 g MPL (Lipamin M20 powder, 5.4% of drink) (2 g MPL/d) Lipamine M20 supplied by: Lecico, GmbH	200 mL/d semi-skimmed milk enriched with cream and skimmed milk concentrate providing 2 g milk fat	TC, LDL-C, HDL-C, TG, PO: TC:HDL-C, Apo A-I, Apo B, CRP, IL-6, sICAM, glucose, insulin, HOMA-IR, SBP, DBP	No	Low risk
Boyle 2019	Boyle et al. [42], 2019 (United Kingdom)	Healthy	Cognitive tests: PL: 26 (100/0) Placebo: 26 (100/0)	21.4 y	<30	Parallel, semi-controlled, intervention: 6-wk	2	250 mL/d milk-based drink enriched with MPL (2.7 g MPL/d) Supplied by: Arla Foods Ingredients, Viby, Denmark	250 mL/d milk-based drink no MPLs	Working memory performance, Executive control function. PO: NR No numerical BP values reported; outcome excluded from synthesis	Yes	Low risk
Vors 2020	Vors et al. [23], 2020 (France) VALOBAB-C study	OW/OB, Abdominal obesity: WC: >80 cm; serum TC <11 mmol/L; serum HDL-C <1.6 mmol/L; serum TG <3 mmol/L	3 g MPL: 19 (0/100) 5 g MPL: 20 (0/100) Control: 19 (0/100)	≤75 y	25–35	Parallel, semi-controlled, intervention: 4-wk, (1-wk run-in)	3	100 g/d cream cheese enriched with buttermilk concentrate-derived 3 g or 5 g MPL/d Buttermilk concentrate supplied by: Candia, Quimper, France	100 g/d full-fat cream cheese, devoid of MPL	PO: TC, LDL-C, HDL-C, TG, TC:HDL-C, Apo A-I, Apo B, Apo B48, Apo B:Apo A-I, Apo B48:Apo B ratio, PCSK9, Glucose, Insulin, HOMA-IR, SBP, DBP, Gut microbiota; SCFA; coprostanol	Yes	Some concerns
Calvo 2023	Calvo et al. [24], 2023 (Spain)	Healthy or mildly cognitively impaired	MFGM: 19 (42/58) Control: 21 (48/52)	>60 y	MFGM: 25.7–29.3 Control: 25.4–29.6	Parallel, placebo-controlled, semi-controlled, intervention: 14-wk	2	200 mL/d MFGM-enriched milk drink (∼0.38 g MPL/d) Supplements produced in collaboration with: INNOLACT S.L., Lugo, Spain and the Aula de Productos Lácteos, APLTA, USC, Spain	200 mL/d skim milk (∼0.001 g PL/d)	TC, LDL-C, HDL-C, TG, Apo A-I, Apo B, Apo B:Apo A-I, glucose, SBP, DBP, cognitive outcomes, PO: NR	No	Some concerns
Zajac 2025	Zajac et al. [25], 2025 (Australia)	Generally healthy^3^ subjective memory complaints (MAC-Q ≥ 25) free from MCI/dementia (MoCA >22)	Completers: Control: 76 Low dose: 75 High dose: 71 sex breakdown NR for completers	55–76 y	18.5—35	Parallel, placebo-controlled, semi-controlled, intervention: 16-wk	3	Skim milk powders fortified with MFGM Phospholipid 100; 50 g/d (two 25 g servings mixed with water) Low-dose MFGM: 1.7 g MPL/d (per 50 g powder) High dose MFGM: 4.0 g MPL/d (per 50 g powder) (50 g powder was mixed with 200 mL water) Supplied by: Fonterra Dairy Co-operative, New Zealand	50 g/d rice starch placebo powder, energy-matched and visually identical, containing 10% whole milk powder (50 g powder was mixed with 200 mL water)	TC, TG, LDL, TNF-α, cognitive function assessed with COMPASS PO: cognitive performance assessed with RBANS	Yes	Some concerns

Abbreviations: Apo A-I, apolipoprotein A-I; Apo B, apolipoprotein B; Apo B100, apolipoprotein B100; Apo B48, apolipoprotein B48; BP, blood pressure; COMPASS, computerized mental performance assessment system; CRP, C-reactive protein; DBP, diastolic blood pressure; F, females; Lp(a), lipoprotein(a); M, males; MAC-Q, memory complaints questionnaire; MCI, mild cognitive impairment; MoCA, Montreal cognitive assessment; MFGM, milk fat globule membrane; MPL, milk polar lipids; NEFA, nonesterified fatty acids; NR, not reported; OB, obese; OW, overweight; PCSK9, proprotein convertase subtilisin/kexin type 9; PL, phospholipid; PO, primary outcome; RBANS, repeatable battery for the assessment of neuropsychological status; RCT, randomized controlled trial; RoB, risk of bias; SBP, systolic blood pressure; sICAM, soluble intercellular adhesion molecule; SCFA, short-chain fatty acids; SL, sphingolipid; SM, sphingomyelin; TC, total cholesterol; TG, triacylglycerol; WC, waist circumference.

1 Fully controlled intervention: all foods and beverages were provided to participants for the duration of the intervention, either for home consumption or consumed on-site (e.g., metabolic ward or research facility). Semi-controlled intervention: experimental foods or supplements were provided to participants, whereas the remainder of the habitual diet was self-selected.

2 Conway et al. [20], 2013 and Conway et al. [28], 2014 represent multiple articles from the same RCT and were assessed as a single study.

3 Baseline HbA1c values across study arms were within the normoglycemic range (<5.7%).

#### Fasting studies

Of the 12 fasting RCTs, 10 studies [18,[21], [22], [23], [24], [25], [26], [27],^42^,^50^] used a parallel design, whereas 2 [19,20] used a crossover design. Intervention durations ranged from 2 to 16 wk, with 5 studies [[18], [19], [20],^23^,^26^] classified as short-term studies and 7 [21,22,24,25,27,42,50] as long-term studies. Eight studies [[18], [19], [20],[24], [25], [26],^42^,^50^] were conducted in healthy adults, and 4 studies [[21], [22], [23],^27^] targeted populations at increased CVD risk. In total, 8 [18,19,21,[24], [25], [26], [27], [28]] studies enrolled both males and females, 3 studies [22,42,50] enrolled males only, and 1 study [23] enrolled females only. Nine RCTs [[18], [19], [20],^22^,[24], [25], [26],^42^,^50^] used supplement-based interventions, including MPL or MFGM-enriched powders, drinks, or tablets supplied by Arla Foods Ingredients, Fonterra Dairy Co-operative, Lecico GmbH, Westland Milk Products, and Avanti Polar Lipids. Two RCTs [21,27] used food-based interventions, including whipping cream or traditional buttermilk. One RCT [23] intervened with a hybrid food–supplement approach by enriching cream cheeses with MPLs derived from freeze-dried buttermilk concentrate. Daily MPL doses ranged from 19.8 mg to 5000 mg with 4 RCTs [20,21,27,50] classified as low dose and 8 [18,19,[22], [23], [24], [25], [26],^42^] as high dose.

#### Postprandial study

The single postprandial study [51] used a crossover design with a 10 to 14 d washout period and evaluated the administration of a standardized high-fat breakfast accompanied by either an MPL-enriched milk drink or an energy- and macronutrient-matched control formulation. The MPL-enriched test drink provided 975-mg milk sphingolipids per serving and was formulated using Lacprodan PL-20 supplied by Arla Foods Amba. Postprandial blood sampling was conducted over a 7-h period after meal consumption in healthy adult males.

### RoB assessment

**FIGURE 2:**
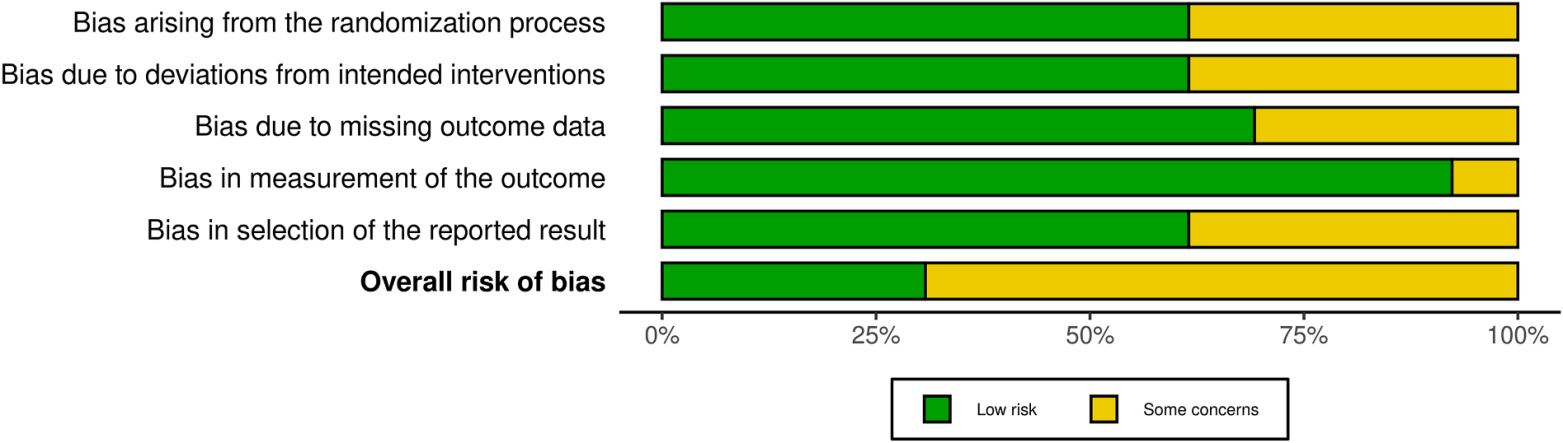
Summary risk-of-bias assessment of randomized controlled trials reporting the impact of milk fat globule membrane and its associated polar lipids on cardiometabolic disease and cognitive function risk markers in the fasted state using the Cochrane Risk-of-Bias tool 2.0

The summary of RoB 2.0 assessment for the included fasting studies is presented in Figure 2, and study-level bias assessment of the included fasting and postprandial trials is presented in Supplemental Figure 1A and B. Among 12 fasting studies (reported across 13 articles), 4 [22,26,27,42] were judged to have an overall low RoB, whereas the remaining 8 [[18], [19], [20], [21],[23], [24], [25],^50^] trials had “some concerns” in ≥1 domain. The single [51] postprandial trial was rated as having “some concerns” overall. Across both fasting and postprandial studies, the domains that most frequently contributed to these concerns were bias arising from the randomization process (D1), bias due to deviations from the intended intervention (D2), and selection of the reported result (D5). Most trials were judged at low risk for measurement of the outcome (D4). No study was rated as being at high RoB in any domain.

### Meta-analysis outcomes

#### Trials comparing MFGM/MPL with control group on fasting blood lipid and lipid-related biomarkers (meta-analysis)

##### TC

**FIGURE 3:**
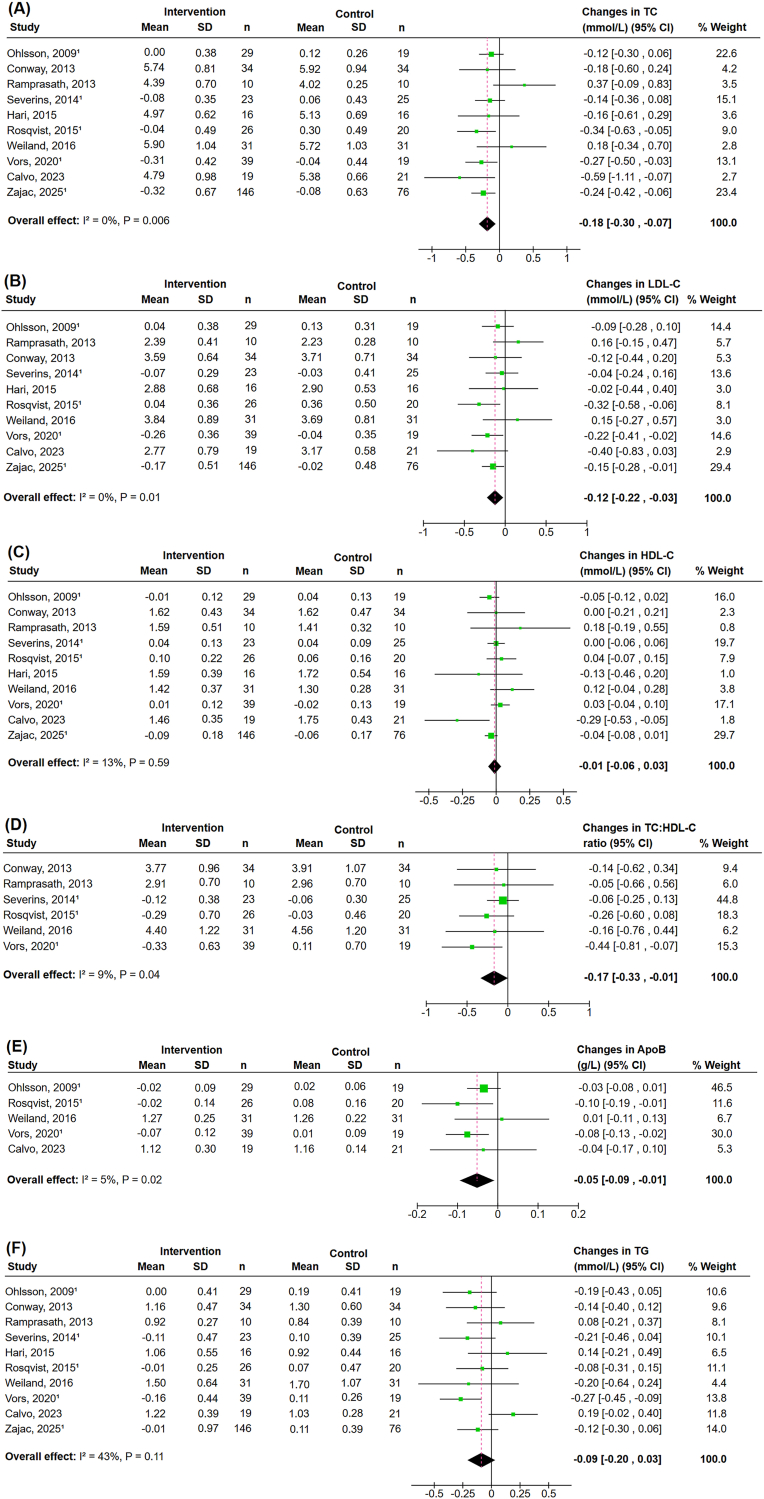
Forest plots of the overall effect of MFGM/MPL on fasting circulating (A) total cholesterol (*n* = 600 participants), (B) LDL cholesterol (*n* = 600 participants), (C) HDL cholesterol (*n* = 600 participants), (D) total cholesterol:HDL cholesterol ratio (*n* = 258 participants), (E) apolipoprotein B (*n* = 254 participants), and (F) triacylglycerol (*n* = 600 participants) in randomized controlled trials. Values were calculated as weighted mean differences (95% CIs) using an inverse-variance random-effects model. The restricted maximum likelihood method was used to estimate heterogeneity variance. The Hartung–Knapp–Sidik–Jonkman correction was applied to estimate the 95% CIs of the summary effects. Horizontal lines represent 95% CIs for individual study estimates; green boxes represent individual study effect estimates, with box size proportional to study weight; the vertical dotted line represents the line of no effect; and black rhombuses represent the pooled effect estimate, with rhombus width indicating the 95% CI. ^1^Change from baseline values were used in analyses. Apo B, apolipoprotein B; CI, confidence interval; MFGM, milk fat globule membrane; MPL, milk polar lipid; TG, triacylglycerol; TC, total cholesterol.

Pooling data from 10 RCTs (*n* = 600 participants) showed that MFGM/MPL intake significantly reduced TC concentrations than the control (WMD: −0.18 mmol/L; 95% CI: −0.30, −0.07; *I*^2^ = 0%) (Figure 3A, Table 3). No significant funnel plot asymmetry was observed (Egger’s test: bias estimate = 0.59, SE = 1.01; *P* = 0.5745), suggesting no evidence of publication bias or small-study effects (Supplemental Figure 2).

**TABLE 3:** Results of meta-analyses of randomized controlled trials comparing the impact of MFGM/MPL supplementation with control groups^1^

Outcome	Number of studies	Participants (*n*)	Results^2^	Heterogeneity
			Meta-analysis overall effect [absolute WMD (95% CI)]	*I*^2^ (%)
Total cholesterol	10 [18–27]	600	−0.18 (−0.30, −0.07) mmol/L	0
LDL cholesterol	10 [18–27]	600	−0.12 (−0.22, −0.03) mmol/L	0
HDL cholesterol	10 [18–27]	600	−0.01 (−0.06, 0.03) mmol/L	13
TC:HDL cholesterol ratio	6 [19–23,27]	258	−0.17 (−0.33, −0.01)	9
Apolipoprotein B	5 [20,22–24,26]	254	−0.05 (−0.09, −0.01) g/L	5
Triacylglycerol	10 [18–27]	600	−0.09 (−0.20, 0.03) mmol/L	43
Apolipoprotein A-I	6 [18,21–24,27]	302	−0.01 (−0.05, 0.04) g/L	18
Apo B:Apo A-I ratio	4 [18,21,23,24]	192	−0.03 (−0.11, 0.04)	67
PCSK9	3 [20,21,23]	138	−12.42 (−76.20, 51.37) μg/L	45
Glucose	5 [21–24,26]	238	−0.03 (−0.18, 0.12) mmol/L	0
Insulin	3 [21–23]	166	−0.80 (−2.12, 0.52) μIU/mL	0
HOMA-IR	3 [21–23]	166	−0.27 (−0.53, −0.00)	0
Systolic blood pressure	5 [22–24,26,28]	226	−0.02 (−4.73, 4.69) mmHg	3
Diastolic blood pressure	5 [22–24,26,28]	226	−1.05 (−3.78, 1.68) mmHg	0
hsCRP	4 [20–22,27]	190	0.08 (−0.41, 0.57) mg/L	0

Abbreviations: CI, confidence interval; hsCRP, high-sensitivity C-reactive protein; MFGM, milk fat globule membrane; MPL, milk polar lipids; PCSK9, proprotein convertase subtilisin/kexin type 9; TC, total cholesterol; WMD, weighted mean difference.

1 Data are presented as WMD (95% CI).

2 Inverse variance random-effects meta-analyses were used to pool effect sizes.

Subgroup analyses are presented in Supplemental Figure 3 and Supplemental Table 4. No statistically significant differences were observed between subgroups when stratified by population health status, intervention duration, or dosage, indicating no evidence of effect modification. All corresponding within-subgroup effects were generally nonsignificant. Although longer-term studies showed a statistically significant reduction (>4 wk; WMD: −0.22 mmol/L; 95% CI: −0.42, −0.03; *I*^2^ = 0%; *n* = 5 trials), the overall analysis did not indicate significant differences by intervention duration.

Sensitivity analyses excluding Zajac et al. [25] slightly attenuated the pooled effect, but it remained significant (WMD: −0.16 mmol/L; 95% CI: −0.30, −0.02; *I*^2^ = 0%; *n* = 9 trials) (Supplemental Table 4). No baseline imbalances were identified in trials with available data; therefore, additional sensitivity analyses were not required.

##### LDL cholesterol

Pooled analyses of 10 RCTs (*n* = 600 participants) showed a significant reduction in LDL cholesterol concentrations after ≥2 wk compared with control (WMD: −0.12 mmol/L; 95% CI: −0.22, −0.03; *I*^2^ = 0%) (Figure 3B, Table 3), with no statistical heterogeneity. There was no significant funnel plot asymmetry (Egger’s test: bias estimate = 0.56, SE = 0.98; *P* = 0.5845), indicating no evidence of publication bias or small-study effects (Supplemental Figure 2).

Subgroup analyses are presented in Supplemental Figure 4 and Supplemental Table 4. No statistically significant differences were observed between subgroups when stratified by baseline health status, intervention duration, or dosage, indicating no evidence of effect modification; within-subgroup effects were nonsignificant.

Sensitivity analyses excluding studies with baseline imbalances [18,24] yielded a pooled effect that remained statistically significant (WMD: −0.12 mmol/L; 95% CI: −0.23, −0.00; *I*^*2*^ = 10%; *n* = 8 trials), whereas exclusion of Zajac et al. [25] rendered the pooled estimate nonsignificant (WMD: −0.11 mmol/L; 95% CI: −0.24, 0.01; *I*^2^ = 17%; *n* = 9 trials) (Supplemental Table 4). However, the direction and magnitude of effect remained broadly consistent, suggesting limited influence of individual studies on the overall findings.

##### HDL cholesterol

Pooled data from 10 RCTs (*n* = 600 participants) showed no meaningful effect of MFGM/MPL on HDL cholesterol (WMD: −0.01 mmol/L; 95% CI: −0.06, 0.03; *I*^2^ = 13%) (Figure 3C, Table 3). Egger’s test showed no significant funnel plot asymmetry (bias estimate = 0.20, SE = 0.77; *P* = 0.7969), indicating no evidence of publication bias or other small-study effects (Supplemental Figure 2).

Subgroup analyses are presented in Supplemental Figure 5 and Supplemental Table 4. A statistically significant difference was observed between baseline health status subgroups (*P* = 0.02), indicating potential effect modification. No statistically significant differences were observed for other subgroup categories (intervention duration or dosage), and within-subgroup effects were nonsignificant.

Sensitivity analyses excluding studies with baseline imbalances [18,24] did not alter the results. Excluding Zajac et al. [25] also did not materially affect the pooled estimate (WMD: −0.00 mmol/L; 95% CI: −0.05, 0.05; *I*^2^ = 9%; *n* = 9 trials) (Supplemental Table 4).

##### TC:HDL cholesterol ratio

Across 6 RCTs (*n* = 258 participants), MFGM/MPL intake produced a statistically significant reduction in the TC:HDL cholesterol ratio than the control (WMD: −0.17; 95% CI: −0.33, −0.01; *I*^2^ = 9%) (Figure 3D, Table 3).

Subgroup analyses are presented in Supplemental Figure 6 and Supplemental Table 4. No statistically significant differences were observed between subgroups when stratified by baseline health status, intervention duration, or dosage, indicating no evidence of effect modification; within-subgroup effects were nonsignificant.

Sensitivity analyses for baseline imbalances were not applicable for this outcome, and Zajac et al. [25] did not report the TC:HDL cholesterol ratio. Therefore, no exclusion-based sensitivity analyses were performed for this outcome.

##### Apo B

Pooled analysis of 5 RCTs (*n* = 254 participants) demonstrated that MFGM/MPL intake produced a statistically significant reduction in apo B concentrations than the control (WMD: −0.05 g/L; 95% CI: −0.09, −0.01; *I*^2^ = 5%) (Figure 3E, Table 3).

Subgroup analyses are presented in Supplemental Figure 7 and Supplemental Table 4. No statistically significant differences were observed between subgroups when stratified by baseline health status or intervention duration, indicating no evidence of effect modification; within-subgroup effects were generally nonsignificant.

No sensitivity analyses relating to baseline imbalances were performed for this outcome, and apolipoprotein data were not reported in Zajac et al. [25]. Therefore, no exclusion-based analyses were conducted.

##### TG

Meta-analysis of 10 RCTs (*n* = 600 participants) showed that MFGM/MPL intake produced a modest, nonsignificant reduction in circulating TG, with moderate heterogeneity (WMD: −0.09 mmol/L; 95% CI: −0.20, 0.03; *I*^2^ = 43%) (Figure 3F, Table 3). No significant funnel plot asymmetry was detected (Egger’s test: bias estimate = 1.19, SE = 1.80; *P* = 0.5278), suggesting no evidence of publication bias or small-study effects (Supplemental Figure 2).

Subgroup analyses are presented in Supplemental Figure 8 and Supplemental Table 4. A statistically significant difference was observed between baseline health status subgroups (*P* = 0.01), suggesting potential effect modification. Among “at risk” participants, a significant reduction was observed (WMD: −0.20 mmol/L; 95% CI: −0.34, −0.06; *I*^2^ = 0%; *n* = 4 trials), whereas no significant effect was observed among healthy participants. All other subgroup analyses (including intervention duration and dose) showed no statistically significant differences between subgroups, and within-subgroup effects for these analyses were nonsignificant.

Sensitivity analyses are reported in Supplemental Table 4. Excluding Ohlsson et al. [18], Severins et al. [27], and Calvo et al. [24] because of baseline imbalances resulted in an attenuated, nonsignificant pooled effect (WMD: −0.11 mmol/L; 95% CI: −0.24, 0.01; *I*^2^ = 15%; *n* = 7 trials). Excluding Zajac et al. [25] did not substantially alter the pooled mean (WMD: −0.08 mmol/L; 95% CI: −0.21, 0.05; *I*^2^ = 48%; *n* = 9 trials).

##### Apolipoprotein A-I

Meta-analysis of 6 RCTs (*n* = 302 participants) showed that MFGM/MPL intake had no meaningful effect on circulating apolipoprotein A-I (apo A-I) concentrations compared with control (WMD: −0.01 g/L; 95% CI: −0.05, 0.04; *I*^2^ = 18%). Overall heterogeneity was low to moderate, and the direction of effects across studies was largely consistent with the pooled estimate (Table 3).

Subgroup analyses are presented in Supplemental Table 4. A statistically significant difference was observed between baseline health status subgroups (*P* = 0.01), suggesting potential effect modification, although no significant effects were observed within individual subgroups. No statistically significant differences were observed for other subgroup categories. No sensitivity analyses were applicable for this outcome.

##### Apo B:apo A-I ratio

Across 4 RCTs (*n* = 192 participants), MFGM/MPL supplementation produced no significant overall effect on the apo B:apo A-I ratio compared with control (WMD: −0.03; 95% CI: −0.11, 0.04; *I*^2^ = 67%), indicating substantial heterogeneity (Table 3). No subgroup analyses or sensitivity analyses were applicable for this outcome.

##### Proprotein convertase subtilisin/kexin type 9

Pooled analysis of 3 RCTs (*n* = 138 participants) produced wide CIs and showed no meaningful overall effect of MFGM/MPL on circulating proprotein convertase subtilisin/kexin type 9 compared with control (WMD: −12.42 μg/L; 95% CI: −76.20, 51.37; *I*^2^ = 45%) (Table 3). No subgroup analyses or sensitivity analyses were applicable for this outcome.

#### Trials comparing MFGM/MPL with control group on fasting markers of glycemic control (meta-analysis)

##### Glucose

**FIGURE 4:**
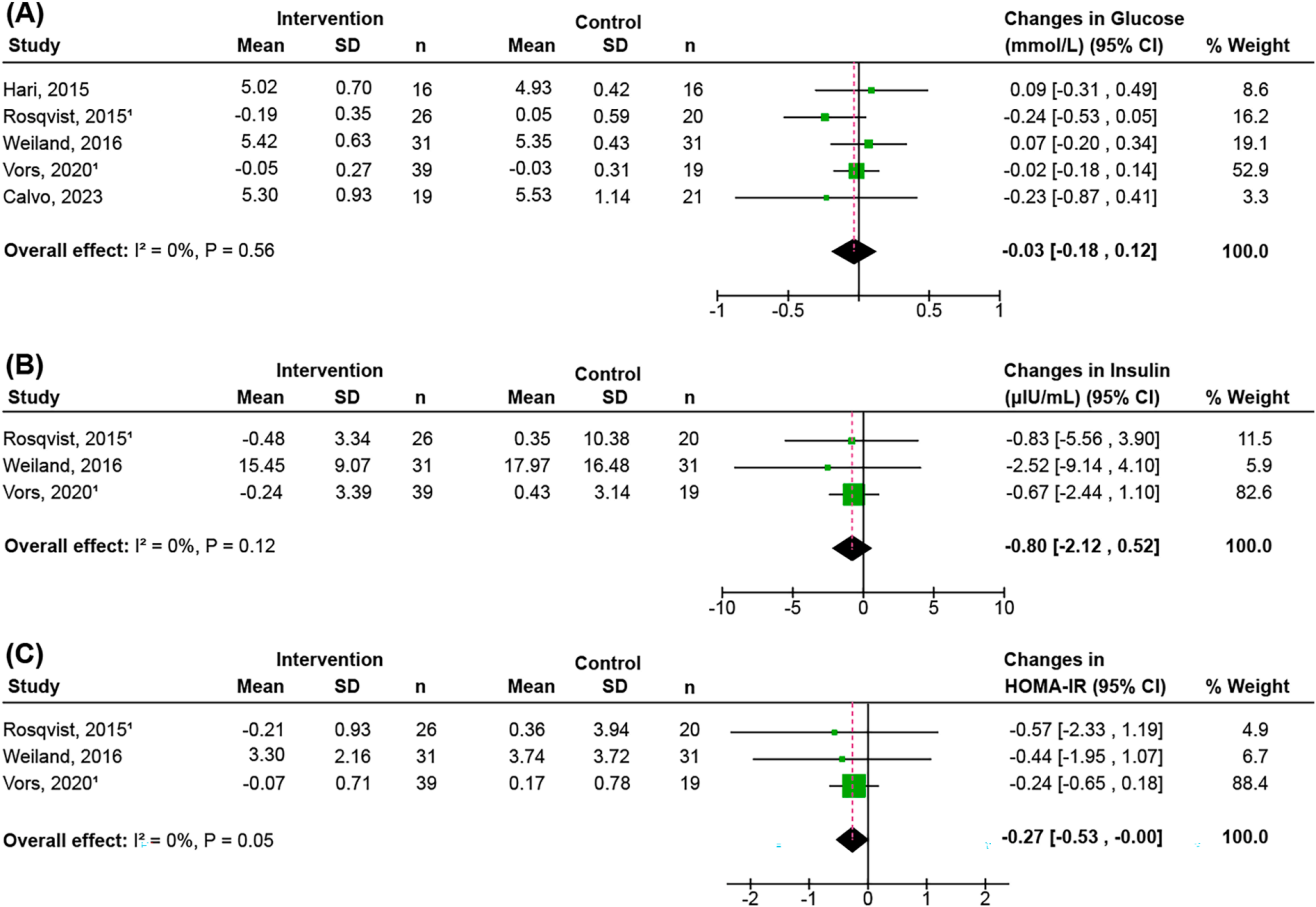
Forest plots of the overall effect of MFGM/MPL fasting circulating (A) glucose (*n* = 238 participants), (B) insulin (*n* = 166 participants), and (C) HOMA-IR (*n* = 166 participants) in randomized controlled trials. Values were calculated as weighted mean differences (95% CIs) using an inverse-variance random-effects model. The restricted maximum likelihood method was used to estimate heterogeneity variance. The Hartung–Knapp–Sidik–Jonkman correction was applied to estimate the 95% CIs of the summary effects. Horizontal lines represent 95% CIs for individual study estimates; green boxes represent individual study effect estimates, with box size proportional to study weight; the vertical dotted line represents the line of no effect; and black rhombuses represent the pooled effect estimate, with rhombus width indicating the 95% CI. ^1^Change from baseline values were used in analyses. CI, confidence interval; MFGM, milk fat globule membrane; MPL, milk polar lipid.

Across 5 RCTs (*n* = 238 participants), MFGM/MPL intake produced no significant effect on fasting glucose concentrations compared with control (WMD: −0.03 mmol/L; 95% CI: −0.18, 0.12; *I*^2^ = 0%) with no statistical heterogeneity, suggesting consistent null effects (Table 3, Figure 4A).

Subgroup analyses are presented in Supplemental Table 4. No statistically significant differences were observed between subgroups when stratified by baseline health status or intervention duration, indicating no evidence of effect modification, and within-subgroup effects were nonsignificant.

Sensitivity analyses were not required for this outcome, as none of the included trials demonstrated baseline imbalances in fasting glucose concentrations, and Zajac et al. [25] did not contribute glucose data.

##### Insulin

Pooled analyses of 3 RCTs involving 166 participants showed that MFGM/MPL supplementation did not significantly affect fasting insulin concentrations compared with control (WMD: −0.80 μIU/mL; 95% CI: −2.12, 0.52), with no statistical heterogeneity (*I*^2^ = 0%) (Table 3, Figure 4B). No subgroup analyses or sensitivity analyses were applicable for this outcome.

##### HOMA-IR

Pooled data from 3 RCTs (*n* = 166 participants) showed a small reduction in HOMA-IR after MFGM/MPL intake compared with control; however, the estimates straddled the null (WMD: −0.27; 95% CI: −0.53, −0.00; *I*^2^ = 0%) (Table 3, Figure 4C). No subgroup or sensitivity analyses were required for this outcome.

#### Trials comparing MFGM/MPL with control group on fasting BP (meta-analysis)

##### Systolic blood pressure

**FIGURE 5:**
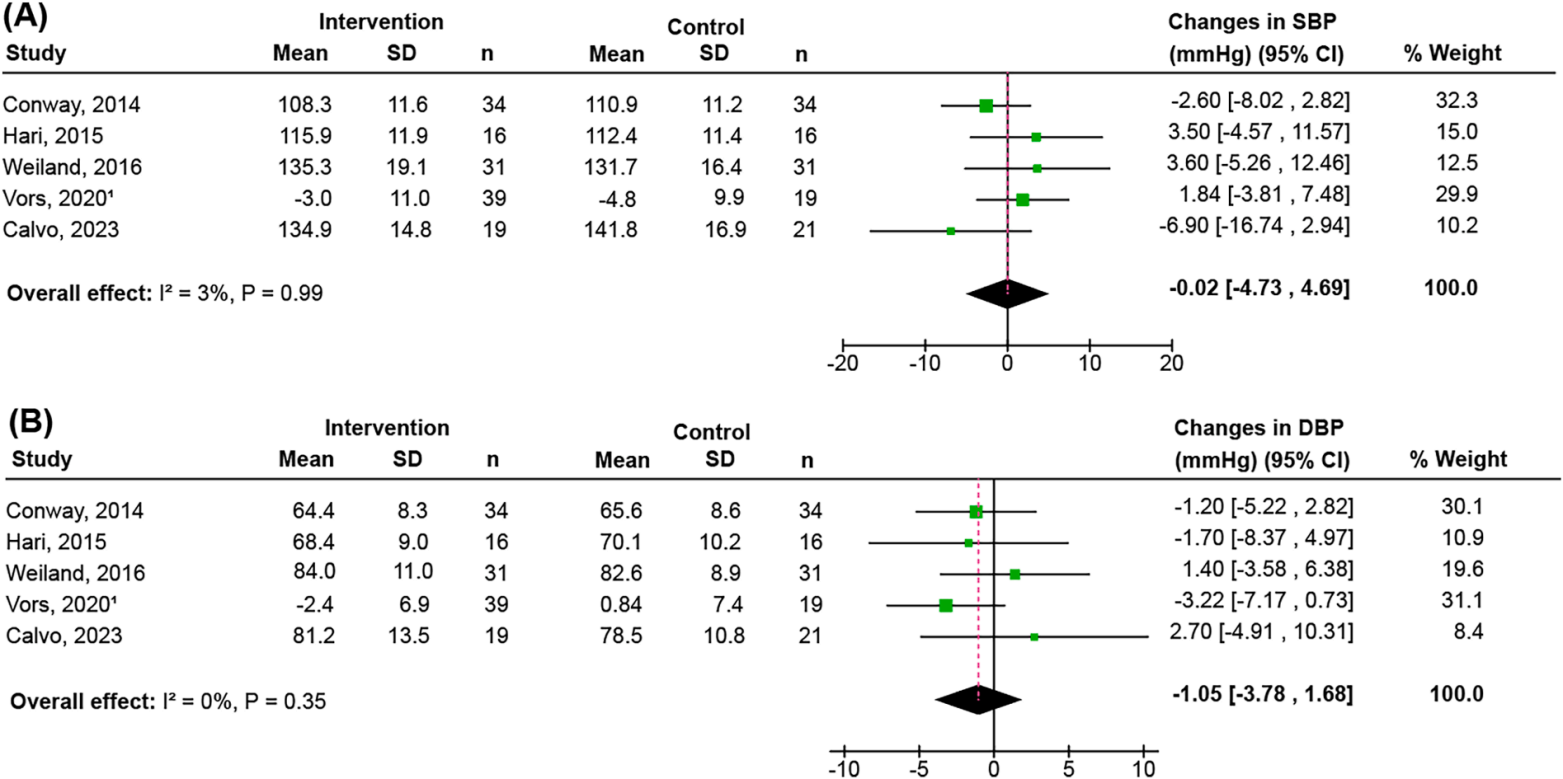
Forest plots of the overall effect of MFGM/MPL (*n* = 226 participants) on fasting (A) systolic and (B) diastolic blood pressure in randomized controlled trials. Values were calculated as weighted mean differences (95% CIs) using an inverse-variance random-effects model. The restricted maximum likelihood method was used to estimate heterogeneity variance. The Hartung–Knapp–Sidik–Jonkman correction was applied to estimate the 95% CIs of the summary effects. Horizontal lines represent 95% CIs for individual study estimates; green boxes represent individual study effect estimates, with box size proportional to study weight; the vertical dotted line represents the line of no effect; and black rhombuses represent the pooled effect estimate, with rhombus width indicating the 95% CI. ^1^Change from baseline values were used in analyses. CI, confidence interval; MFGM, milk fat globule membrane; MPL, milk polar lipid; SBP, systolic blood pressure; DBP, diastolic blood pressure.

Pooling data from 5 RCTs (*n* = 226 participants) showed no significant effect of MFGM/MPL intake on systolic blood pressure (SBP) when compared with control (WMD: −0.02 mmHg; 95% CI: −4.73, 4.69; *I*^2^ = 3%) (Table 3, Figure 5A).

Subgroup analyses are presented in Supplemental Table 4. No statistically significant differences were observed between subgroups when stratified by baseline health status or intervention duration, indicating no evidence of effect modification; within-subgroup effects were nonsignificant. Sensitivity analyses were not applicable for this outcome.

##### Diastolic blood pressure

Pooled analysis of 5 RCTs (*n* = 226 participants) showed no significant effect of MFGM/MPL supplementation on diastolic blood pressure (DBP) compared with control (WMD: −1.05 mmHg; 95% CI: −3.78, 1.68; *I*^2^ = 0%) (Table 3, Figure 5B).

Subgroup analyses are presented in Supplemental Table 4. A statistically significant difference was observed between intervention duration subgroups (*P* < 0.0001), with high between-subgroup heterogeneity (*I*^2^ = 95%). However, effects within both short- and long-term subgroups were not statistically significant. No significant differences were observed for baseline health status, and within-subgroup effects were also nonsignificant. Overall subgroup differences were statistically significant (*P* = 0.0002), with substantial heterogeneity across subgroup comparisons (*I*^2^ = 85%). Sensitivity analyses were not applicable for this outcome.

#### Trials comparing MFGM/MPL with control group on fasting inflammatory markers (meta-analysis)

##### High-sensitivity C-reactive protein

Pooling data from 4 RCTs (*n* = 190 participants) showed that MFGM/MPL intake did not meaningfully affect circulating high-sensitivity C-reactive protein concentrations compared with control (WMD: 0.08 mg/L; 95% CI: −0.41, 0.57; *I*^2^ = 0%) (Table 3). No subgroup or sensitivity analyses were required for this outcome.

### Narrative synthesis outcomes

#### Trials comparing MFGM/MPL with control group on other fasting blood lipid and lipid-related biomarkers (narrative synthesis)

Of the included trials, 4 RCTs reported additional fasting blood lipid or related outcomes when comparing MFGM/MPL interventions with control (Supplemental Table 5A). Rosqvist et al. [21], comparing an 8-wk MFGM diet of whipping cream enriched with MFGM (19.8 mg MPL/d) with the control diet, found no significant difference between groups in the fasting LDL:HDL ratio and VLDL cholesterol concentrations. However, changes in circulating non-HDL cholesterol differed significantly between groups and favored the MFGM diet, with concentrations decreasing in the MFGM group (mean ± SD: −0.14 ± 0.51 mmol/L) and increasing in the control group (mean ± SD: 0.24 ± 0.49 mmol/L; *P* = 0.013) [21]. In line with Rosqvist et al. [21], Ramprasath et al. [19] did not find significant differential effects on VLDL cholesterol concentrations following 2-wk consumption of controlled diets with or without 1 g/d SM. The RCT by Ohlsson et al. [18], where participants consumed an MPL-enriched buttermilk drink (2.8 g MPL/d) or a control drink for 4 wk, found no differences in fasting lipoprotein(a) concentrations. Vors et al. [23], who compared cream cheeses containing 3 g or 5 g MPL/d for 4 wk with a control cream cheese, observed that apolipoprotein B48 (apo B48) concentrations were significantly reduced in the 5 g MPL/d group (mean ± SEM: −2.04 ± 0.50 μg/mL) than in the control (mean ± SEM: 0.41 ± 0.36 μg/mL) and 3 g MPL/d (mean ± SEM: −0.13 ± 0.39 μg/mL) groups (*P* = 0.0004). Consistently, the apo B48:apo B ratio was also significantly reduced in the 5 g MPL group (mean ± SEM: −1.64 ± 0.45) than in the control and 3 g MPL groups (mean ± SEM: 0.38 ± 0.45 and 0.14 ± 0.47, respectively; *P* = 0.005). However, in the crossover RCT by Conway et al. [20], 4-wk supplementation with a buttermilk drink rich in MFGM components (187.5 mg MPL/d) resulted in a modest reduction in apo B48 concentrations (−12.9%) that did not reach statistical significance (*P* = 0.195). No significant changes in fasting apolipoprotein B100 (apo B100) concentrations were observed compared with placebo, consistent with findings from the 4-wk RCT by Severins et al. [27], which also reported no significant differences in change from baseline in apo B100 concentrations after consumption of 80 mL/d of traditional buttermilk containing 0.09% MPL compared with a skimmed milk control. In the RCT by Hari et al. [26] comparing MFGM tablets providing 924 mg MPL/d with a placebo found no between-group differences in fasting nonesterified fatty acid concentrations. RoB was rated as “some concerns” for all trials in this subsection, except for Severins et al. [27] and Hari et al. [26], which were rated as low risk.

#### Trials comparing MFGM/MPL with control group on fasting inflammatory markers (narrative synthesis)

Two RCTs reported additional inflammatory marker outcomes comparing MFGM/MPL interventions with control (Supplemental Table 5B). In an 8-wk intervention, Weiland et al. [22] compared semi-skimmed milk enriched with 2 g MPL with a control milk and found no significant between-group differences in IL-6 or soluble intercellular adhesion molecule-1 concentrations (RoB: low risk). In the 16-wk intervention, Zajac et al. [25] compared a powdered product providing 1.7 g MPL/d or 4 g MPL/d with a matched control product. In the per-protocol analyses, the authors reported a favorable treatment effect on TNF-α (change from baseline: control: 0.95 pg/mL; 95% CI: −5.02, 6.92; low dose: −3.37 pg/mL; 95% CI: −9.3, 2.55; high dose: −5.34 pg/mL; 95% CI: −13.44, 2.75; *P* = 0.008), with greater reductions observed in the high dose group (high dose vs. control *P* = 0.06). The intention-to-treat analysis showed a similar pattern but did not reach statistical significance (change from baseline: control: 0.59 pg/mL; 95% CI: −3.27, 4.47; low dose: −2.22 pg/mL; 95% CI: −6.16, 1.72; high dose: −3.21 pg/mL; 95% CI: −7.70, 1.28; *P* = 0.07) (RoB: some concerns).

#### Trials comparing MFGM/MPL with control group on gut microbiome and fecal metabolic outcomes (narrative synthesis)

One RCT [23] reported gut-related outcomes (Supplemental Table 5C). The authors found that 4-wk supplementation with MPL (3 g/d or 5 g/d) did not differentially affect the abundance of the major phylogenetic groups of the gut microbiota, including *Firmicutes*, *Bacteroides-Prevotella*, *Akkermansia muciniphila*, *Bifidobacterium* spp., *Lactobacillus–Leuconostoc–Pediococcus*, *Clostridium coccoides*, *Clostridium leptum*, *Faecalibacterium prausnitzii*, *Roseburia–Eubacterium rectale*, *Veillonella* spp., the *Enterobacteriaceae* family, *Escherichia coli*, and *Bilophila wadsworthia*, nor the fecal short-chain fatty acid profile, compared with control. Fecal coprostanol concentrations tended to differ between groups (*P* = 0.07) and were significantly higher in the MPL groups than in the control group (*P* = 0.03). The fecal coprostanol:cholesterol ratio was significantly increased after MPL intake (*P* = 0.04) (RoB: some concerns).

#### Trials comparing MFGM/MPL with control group on cognitive function outcomes

Four trials reported cognitive outcomes when comparing MFGM/MPL interventions with control (Supplemental Table 5D). In the RCT by Zajac et al. [25], participants consumed either a powdered product providing 1.7 g MPL/d or 4 g MPL/d for 16 wk compared with a matched control product. The authors reported no significant effects of the intervention on repeatable battery for the assessment of neuropsychological status (RBANS) scores or computerized mental performance assessment system (COMPASS) cognitive domain performance, with all CIs including zero. Schubert et al. [50], in a 12-wk RCT, intervened with beverages containing 0.5% MPL (150 mg/d), 1% MPL (300 mg/d), or a control beverage, found no main effects of treatment on memory performance. However, after an acute stress test, visual memory performance improved significantly in older adults consuming the higher MPL dose compared with the low dose and control groups (*P* = 0.042). Boyle et al. [42], comparing an MPL drink providing 2.7 g MPL/d with a control drink for 6 wk, observed no between-group differences in n-back or attention-switch performance post-intervention. However, participants in the MPL treatment arm demonstrated lower repeat-trial performance costs (faster reaction time) relative to placebo (*P* = 0.01). Calvo et al. [24], who compared a 14-wk intervention of MFGM-enriched milk drink containing 0.38 g MPL/d with a control milk drink, found no overall effect of treatment on global cognitive domains; however, short-delay cued recall improved in females receiving MFGM compared with control (*P* = 0.044), with no effect observed in males. In this subsection, RoB was rated as “some concerns” for all trials except Boyle et al. [42], which was rated as low risk.

#### Trial comparing acute (postprandial) intake of MFGM/MPL with control group on cardiometabolic outcomes (narrative synthesis)

Among the included studies, 1 RCT evaluated the postprandial effects of a sphingolipid-enriched dairy formulation containing 975 mg milk sphingolipids, ingested with a standardized meal, compared with a matched control drink in healthy adults [51] (see Supplemental Table 6). Ohlsson et al. [51] reported that postprandial responses were largely identical between treatments, with no significant differences in TG, insulin, glucose, apo A-1, or apo B concentrations over the 7-h postprandial period. TC concentrations were slightly lower after the sphingolipid-enriched drink, although this difference did not reach significance. Postprandial decreases in HDL and LDL cholesterol at 1 to 3 h were more pronounced after the control drink; however, these differences were not significant in paired analyses. The iAUC for cholesterol in TG-rich lipoproteins was 30% lower after the sphingolipid-enriched drink, although the difference did not reach statistical significance, with only a nonsignificant trend toward lower cholesterol in TG-rich lipoproteins at 1 h postprandially (*P* = 0.063) (RoB: some concerns).

### Certainty of evidence

GRADE evidence profiles for the effects of MFGM/MPL consumption on critical fasting CMD risk markers are presented in Table 4 and Supplemental Table 7. The certainty of evidence was moderate for most blood lipid outcomes, including TC, LDL cholesterol, apo B, and the TC:HDL cholesterol ratio, but was low for HDL cholesterol and TG concentrations. Certainty was also low for glycemic markers (glucose, insulin, and HOMA-IR) and for BP outcomes. The RoB domain was downgraded by one level to serious for all outcomes because of the proportion of studies rated as having “some concerns” and their contribution to the pooled effect estimates. Inconsistency was judged as not serious, although variation in interventions and comparators across RCTs was noted. Despite this, the direction and magnitude of effects were sufficiently consistent to support a single pooled estimate. Heterogeneity was minimal (*I*^2^ < 50%), supporting the decision not to downgrade for inconsistency. Indirectness was judged as not serious across all outcomes as the study populations, exposures, and comparators were relevant to the review question. Imprecision was judged as not serious for most blood lipid outcomes (total, LDL cholesterol, TC:HDL cholesterol ratio, and apo B). However, HDL cholesterol, TG, glycemic markers, and BP outcomes were downgraded one level because the 95% CI crossed the threshold of important benefit or harm, including the possibility of no effect.

**TABLE 4:** GRADE evidence profile for the effects of MFGM/MPLs on critical fasting cardiometabolic disease risk markers in disease-free adults^1^

Certainty assessment					
Total studies (references)	Outcome	No of participants (studies)	Effect [absolute WMD (95% CI)]	Certainty	Importance of outcome
Blood lipids					
10 [[18], [19], [20], [21], [22], [23], [24], [25], [26], [27]]	TC	600 (10 RCTs)	−0.18 (0.30, −0.07) mmol/L	⊕⊕⊕◯ Moderate	Critical
10 [[18], [19], [20], [21], [22], [23], [24], [25], [26], [27]]	LDL-C	600 (10 RCTs)	−0.12 (0.22, −0.03) mmol/L	⊕⊕⊕◯ Moderate	Critical
10 [[18], [19], [20], [21], [22], [23], [24], [25], [26], [27]]	HDL-C	600 (10 RCTs)	−0.01 (−0.06, 0.03) mmol/L	⊕⊕◯◯ Low	Critical
6 [[19], [20], [21], [22], [23],^27^]	TC:HDL-C ratio	258 (6 RCTs)	−0.17 (−0.33, −0.01)	⊕⊕⊕◯ Moderate	Critical
5 [18,[21], [22], [23], [24]]	Apo B	254 (5 RCTs)	−0.05 (−0.09, −0.01) g/L	⊕⊕⊕◯ Moderate	Critical
10 [[18], [19], [20], [21], [22], [23], [24], [25], [26], [27]]	TG	600 (10 RCTs)	−0.09 (−0.20, 0.03) mmol/L	⊕⊕◯◯ Low	Critical
Glycemic control					
5 [[21], [22], [23], [24],^26^]	Glucose	238 (5 RCTs)	−0.03 (−0.18, 0.12) mmol/L	⊕⊕◯◯ Low	Critical
3 [[21], [22], [23]]	Insulin	166 (3 RCTs)	−0.80 (−2.12, 0.52) μIU/mL /L	⊕⊕◯◯ Low	Critical
3 [[21], [22], [23]]	HOMA-IR	166 (3 RCTs)	−0.27 (−0.53, −0.00)	⊕⊕◯◯ Low	Critical
Blood pressure					
5 [[22], [23], [24],^26^,^28^]	SBP	226 (5 RCTs)	−0.02 (−4.73, 4.69) mmHg	⊕⊕◯◯ Low	Critical
5 [[22], [23], [24],^26^,^28^]	DBP	226 (5 RCTs)	−1.05 (−3.78, 1.68) mmHg	⊕⊕◯◯ Low	Critical

Abbreviations: Apo B, apolipoprotein B; CI, confidence interval; DBP, diastolic blood pressure; GRADE, Grading of Recommendations, Assessment, Development, and Evaluation; MFGM, milk fat globule membrane; MPL, milk polar lipid; RCT, randomized controlled trial; SBP, systolic blood pressure; TC, total cholesterol; TG, triacylglycerol; WMD, weighted mean difference.

1 The GRADE approach categorizes outcomes by importance. Outcomes rated as “critical” are considered most important for informing public health guidelines and policy [37].

## Discussion

To our knowledge, this is the first SRMA to provide a comprehensive, harmonized evaluation of bovine-derived MFGM/MPL effects across fasting and postprandial cardiometabolic markers (including blood lipids, apolipoproteins, glycemic markers, and BP), emerging cardiometabolic biomarkers, and cognitive outcomes in adults. Building on the single prior SRMA [31], which focused on fasting lipid outcomes and included 1 multicomponent intervention, our review isolates the specific physiologic effects of MFGM/MPL by synthesizing evidence exclusively from isoenergetic comparator trials without co-interventions. We demonstrated that 2 to 16 wk of MFGM or MPL intake (19.8–5000 mg/d) produced modest but consistent overall improvements in fasting lipid and apolipoprotein profiles, particularly TC, LDL cholesterol, apo B, and the TC:HDL cholesterol ratio, with low between-study heterogeneity and moderate certainty of evidence. In contrast, evidence for fasting HDL cholesterol, TG, glycemic markers, and BP showed no clear overall effects, and certainty was low, constraining the strength of inference for these outcomes. As highlighted in our narrative synthesis, few studies were designed specifically to interrogate emerging cardiometabolic risk biomarkers, such as inflammatory markers, gut microbiome- or fecal-metabolism-related outcomes, or postprandial responses, and cognitive function, limiting the strength of inference for these outcomes.

Our findings extend the prior SRMA of 6 RCTs that reported cholesterol-lowering effects of MFGM, delivered alone or (in 1 study) alongside a multicomponent intervention [31]. Pooling 10 RCTs, we observed a mean LDL cholesterol reduction of 0.12 mmol/L relative to control. Although the absolute LDL cholesterol reduction is modest, shifts of this magnitude can still be meaningful at the population level, given evidence from a large prospective meta-analysis of 14 statin trials (90,056 participants) that a 1.0 mmol/L decrease in LDL cholesterol is associated with a ≈19% lower risk of coronary mortality [59]. This contextualizes the potential cardiometabolic relevance of MFGM/MPL, even when their effects are smaller than those achieved with pharmacological therapy. Importantly, pooled reductions in TC and LDL cholesterol were consistent across trials, with heterogeneity estimated at *I*^2^ = 0%, despite minor between-study differences in intervention characteristics, including dose and duration.

Mechanistic and comparative evidence support a biologically plausible basis for these lipid effects. A 2024 SRMA of supplemental SM interventions from milk, soy, and egg sources [60] reported reductions in total and LDL cholesterol concentrations in adults without metabolic syndrome [60]. Notably, milk-derived MPLs are uniquely rich in SM (∼23%–25% of total MPL), whereas egg and soy phospholipids contain ≤3% SM [14,61,62]. MFGM-derived MPLs, particularly SM, are characterized by long-chain saturated acyl groups and diverse sphingoid bases that can form ordered complexes with relatively high affinity for cholesterol, thereby limiting micellar solubilization and intestinal absorption [63,64]. In contrast, the shorter and more unsaturated fatty acyl chains that predominate in nondairy phospholipids interact less strongly with cholesterol and are therefore less effective at inhibiting its absorption [63,64]. Beyond effects on micellar processing, the distinct structural and metabolic profile of milk-derived SM, relative to other dietary SM sources, also contributes to favorable shifts in circulating sphingolipids, of which SM is a major subclass, as well as downstream ceramide species [65]. Human data from an RCT in our SRMA [23], together with complementary evidence from the studies by Le Barz et al. [65] and Sprenger et al. [66] indicate that MFGM/MPL-induced improvements in circulating lipids (total, LDL cholesterol, and apo B) are associated with reductions in atherogenic SM and ceramide species. Preclinical and in vitro studies support this mechanism, showing that SM-rich MPL can reduce intestinal cholesterol absorption, increase fecal sterol excretion, and subsequently lower circulating and hepatic cholesterol pools [63,64,67,68]. Human evidence reinforces reduced intestinal absorption as a key mechanism. In the VALOBAB-C RCT [23], conducted in postmenopausal females at elevated CVD risk, 4-wk MPL supplementation (5 g/d) reduced postprandial intestine-derived TG-rich lipoproteins and decreased chylomicron-rich fraction particle number. Consistent with this, the complementary acute VALOBAB-D crossover study [23] in ileostomy patients using deuterium-labeled cholesterol showed reduced appearance of labeled cholesterol in chylomicrons and increased ileal cholesterol efflux following MPL-containing meals. Approximately 20% to 25% of ingested milk SM was recovered intact in ileal efflux, suggesting incomplete intestinal absorption due to the formation of unabsorbed SM-cholesterol complexes [23]. Together, these converging findings strengthen the biological plausibility that MFGM-derived MPLs modulate lipid metabolism predominantly via effects on intestinal cholesterol handling and downstream sphingolipid pathways.

The inclusion of apolipoprotein markers as outcomes is a key strength of this SRMA. In particular, apo B, the structural protein of all major atherogenic lipoproteins (LDL, VLDL, intermediate-level lipoproteins, and remnants), provides a direct measure of atherogenic particle number independently of cholesterol content [69]. Although LDL cholesterol remains the primary therapeutic target for atherosclerotic CVD prevention, apo B is increasingly recognized as a more accurate indicator of CVD risk than TC or LDL cholesterol [69]. Although the pooled reduction in circulating apo B was modest, the overall pattern of effects was directionally consistent, with most trials showing apo B reduction and heterogeneity remaining very low, which suggests that MFGM/MPL intake may preferentially influence apo B-containing atherogenic lipoproteins. Consistent with this pattern, a null overall effect on circulating HDL cholesterol was observed. An exception was reported by Ramprasath et al. [19], who observed an increase in HDL cholesterol following short-term supplementation with milk SM. Similarly, the VALOBAB-C trial [23] reported a dose-specific increase in HDL cholesterol concentrations in the 5 g/d MPL group than in the 3 g/d MPL and control groups. However, because the 2 MPL doses were combined for meta-analysis, this isolated increase was diluted and did not affect the overall pooled estimate. The absence of a pooled effect on apo A-I (the major structural/functional protein of HDL particles [70]) across trials further supports that the observed improvement in the TC:HDL cholesterol ratio is driven primarily by reductions in TC and LDL cholesterol, rather than changes in HDL cholesterol.

Although no significant overall effect of MFGM/MPL intake on fasting TG was observed, subgroup analyses indicated that reductions were more apparent in participants with elevated CVD risk. Although subgroups were defined as “healthy” or “at risk” based on overweight/obesity or elevated LDL cholesterol rather than baseline TG, individuals with greater cardiometabolic risk often exhibit higher TG concentrations and responses, providing more physiologic scope for improvement [[71], [72], [73]]. This observation should be interpreted cautiously, given the limited number of contributing studies, low certainty of evidence, and moderate heterogeneity for TG (*I*^2^ = 42%). This pattern is biologically plausible because fasting TG is more sensitive than other evaluated lipids, such as LDL cholesterol, to adiposity, insulin resistance, and broader metabolic dysregulation [74,75].

In contrast, no significant pooled effect was observed for SBP or DBP. The certainty of evidence was low; BP was not a primary outcome of the included trials, and participants were generally normotensive, reducing the likelihood of detecting a BP-lowering effect. It is, however, noteworthy that 4-wk consumption of buttermilk (187.5 mg MPL/d) led to significant reductions in systolic and arterial BP, alongside lower plasma angiotensin-converting enzyme (ACE) concentrations, in normotensive adults [28]. Given the central role of ACE in the renin-angiotensin system, these findings suggest a biologically plausible mechanism through which MFGM/MPL may exert BP-lowering effects [76]. Trials in hypertensive populations, powered for BP endpoints, are warranted.

Subgroup analyses across outcomes suggested potential effect modification by baseline health status for TG and HDL cholesterol and by intervention duration for DBP; however, these findings should be interpreted cautiously given the limited number of contributing studies and, in some cases, substantial heterogeneity across estimates.

Similarly, MFGM/MPL intake did not meaningfully affect fasting glucose or insulin concentrations. A small, borderline improvement in HOMA-IR was observed, but this was based on few studies and low certainty and should be interpreted cautiously. Mechanistically, improved insulin sensitivity remains plausible via favorable modulation of ceramide species, reduced postprandial secretion of TG-rich lipoproteins, lower hepatic lipid burden via suppressed intestinal cholesterol absorption, and potential anti-inflammatory or gut-mediated effects (for review, see [17]). Larger and longer-duration RCTs in “at-risk” cohorts are required to determine whether these mechanistic signals translate into clinically relevant changes.

Individuals spend a large proportion of the day in the non-fasting (postprandial) state, and elevated postprandial TG concentrations are directly associated with increased CVD risk [77]. However, evidence on postprandial responses to MFGM/MPL intake remains limited. Only 1 eligible acute crossover study by Ohlsson et al. [50] met inclusion criteria, reporting similar 7-h postprandial TG, cholesterol, apo B, apo A-I, insulin, and glucose responses following an MPL-rich dairy formulation vs. a control product. Several other RCTs [56] have reported postprandial cardiometabolic responses after MFGM- or MPL-containing meals, but were excluded from our SRMA based on prespecified methodological criteria. Postprandial analyses from the VALOBAB-C study suggested reductions in postprandial TC, TG, apo B:apo A-I ratio, and apo B48 response after 4-wk intake of higher-dose MPL (5 g/d) [23], but these acute-within-chronic data were not eligible for inclusion.

Evidence relating to cognitive outcomes spanned 4 RCTs [24,25,42,50] but was too heterogeneous for robust pooling, reflecting a wide variation in assessment tools, scoring, domains, and reporting approaches. These outcomes were therefore synthesized narratively in accordance with Cochrane guidance [41]. Although isolated domain-specific improvements were reported in some RCTs, including memory under acute stress [50], faster reaction-time performance [42], and short-delay cued recall in females [24], effects were inconsistent and insufficient to indicate a general cognitive benefit. Certain cognitive processes may be selectively responsive to MFGM/MPL intake and merit further study. Well-designed RCTs using standardized cognitive domains, validated digital batteries, and complementary physiologic markers are needed to evaluate these effects more rigorously. Although these findings support emerging interest in the potential neurocognitive relevance of MFGM/MPLs [30], the current evidence base remains too limited and methodologically heterogeneous to support definitive interpretation.

A key strength of this SRMA is its comprehensive assessment of traditional and emerging CVD risk markers, alongside postprandial, mechanistic, and cognitive outcomes, within a unified evidence framework. This policy-relevant synthesis adhered to Cochrane-recommended methods for RoB assessment [39] and applied the GRADE framework [37] to critical outcomes to generate transparent, decision-ready estimates of evidence certainty, enabling interpretation and use within nutrition guideline development contexts. Subgroup and sensitivity analyses enhanced interpretability by exploring potential sources of heterogeneity. The use of strict PICOS criteria ensured the isolation of the specific effects of MFGM/MPL by requiring isoenergetic comparators without co-interventions, minimizing confounding from nutrient displacement or concurrent dietary or lifestyle modifications. In addition, statistical procedures followed the best practice for SRMAs with relatively small numbers of contributing trials, with heterogeneity estimated using the restricted maximum likelihood method and summary effects derived using the HKSJ adjustment [41,78].

Several limitations should also be acknowledged. The literature search was restricted to 3 electronic databases and did not include gray literature, which may have increased risk of publication bias. However, funnel plot assessment did not indicate asymmetry for traditional lipid outcomes. In 1 included trial [25], individual-level diabetes status and glucose-lowering medication use were not reported, and analyses restricted to participants without diabetes were unavailable. Although the trial eligibility criteria permitted the inclusion of individuals with medically managed type 2 diabetes above specified HbA1c thresholds, baseline HbA1c values were within the normoglycemic range with low variability across study arms, suggesting a relatively homogeneous metabolic phenotype. Furthermore, sensitivity analyses excluding this study did not materially alter pooled effect estimates, supporting the robustness of the findings. Nevertheless, future trials would benefit from reporting individual-level disease status and medication use or from providing subgroup analyses to further strengthen interpretability in disease-free populations. Meta-analysis was not feasible for several outcomes because of the limited number of available studies and heterogeneity, necessitating narrative synthesis. In addition, subgroup analyses were exploratory and sometimes contained few studies, limiting statistical power and precision; these should be viewed as hypothesis generating rather than definitive. Finally, most included studies delivered relatively large supplemental doses of MFGM/MPL that exceed amounts typically obtainable from habitual dairy consumption [12], which constrains generalizability to whole-food contexts. Based on mean United Kingdom adult dairy intakes from the national diet and nutrition survey (NDNS) and reported MPL concentrations in milk (9.4–35.5 mg/100 g) [79,80], habitual dietary MPL intake is ∼20 to 90 mg/d, substantially lower than the doses used in supplement-based RCTs (∼150–5000 mg/d). Nevertheless, RCTs such as those by Zajac et al. and Vors et al. [23,25] indicate that intakes of 4 to 5 g of MPL/d can be safely incorporated into habitual diets without adverse effects, supporting the practical feasibility of targeted supplementation. Taken together, although higher supplemental doses may limit relevance to everyday dietary patterns, the available evidence points to a potential role for MFGM/MPL as nutraceutical or functional food ingredients for modulating atherogenic lipoprotein metabolism and broader cardiometabolic risk markers.

## Recommendations for future research

Most included RCTs were short in duration (2–8 wk), with only 2 trials lasting 14 to 16 wk. Longer-term, well-designed RCTs are needed to evaluate the effects of MFGM and its components on emerging cardiometabolic risk markers and cognitive function, particularly in middle-to-older aged cohorts at risk of CVD and dementia. Future trials should incorporate advanced lipid and lipoprotein phenotyping, including LDL particle size and subclass distribution, which may offer more sensitive indicators of cardiometabolic risk than traditional lipid measures [81,82]. Although no included studies have reported such measures, our recently conducted registered RCT (NCT05939544) is expected to contribute to this evidence gap.

Beyond lipid metabolism, further research should explore whether MFGM/MPL supplementation can influence vascular aging, including outcomes such as arterial stiffness, an established predictor of both CVD and cognitive decline [[83], [84], [85]]. Emerging evidence also supports potential interactions between the gut–brain axis, including modulation of gut barrier integrity, systemic inflammation, and gut-derived metabolites such as short-chain fatty acids [17,23,65]. Integrated human RCTs that combine cardiometabolic, vascular, gut, and cognitive outcomes would help clarify these pathways.

Methodologically, future postprandial studies should ensure that MFGM/MPL is the sole differing component in test meals, as several potentially relevant RCTs were excluded from this SRMA due to confounding differences in fat type or meal composition. Given the prolonged TG excursion, future studies should monitor postprandial lipemia for ≥6 to 8 h [77], with frequent sampling to capture the peak TG response [77].

Considerable variability also exists in intervention format, dose, and ingredient composition (for detailed review, see [16]). Producing consistent, high-purity MFGM isolates remains challenging, particularly with respect to isolating individual MFGM fractions, and commercially available MFGM-enriched ingredients vary considerably in the levels of major components [16]. Standardized reporting and compositional characterization are needed to improve comparability between trials and support clearer interpretation of which components drive observed effects.

## Conclusions

In conclusion, this SRMA provides up-to-date evidence that intake of bovine-derived MFGM or membrane-associated MPLs modestly improves fasting circulating atherogenic lipid markers, specifically LDL cholesterol and apo B, in disease-free adults. These findings highlight the potential cardiometabolic relevance of these bioactive components, although most included trials delivered MFGM/MPLs in isolated supplement or ingredient formats. As conventional dairy products contain much lower quantities of MFGM/MPLs than the doses used in supplement-based trials, and because these components are present within more complex food matrices, the extent to which these effects translate to whole-food contexts remains uncertain. Evidence for emerging fasting cardiometabolic biomarkers, including inflammatory markers, microbiome- and gut-related outcomes, postprandial cardiometabolic responses, as well as cognitive effects also remains limited, highlighting key evidence gaps. Taken together, these findings provide, to our knowledge, the most comprehensive synthesis to date indicating that MFGM/MPL components may exert modest but physiologically meaningful improvements in atherogenic lipid profiles, while identifying key priorities for future RCTs and mechanistic studies. Further well-designed, adequately powered, longer-term trials are needed to determine clinical significance and clarify the relevance of these findings for both supplemental and functional-food applications incorporating MFGM/MPL.

## Author contributions

The authors’ responsibilities were as follows – ASB: performed the literature searches and conducted the meta-analyses; ASB, OM: designed the systematic review protocol and database search strategy, contributed to the data analysis plan, performed synthesis, data harmonization, grading of evidence, and wrote the manuscript; ASB, YT, OM: contributed to the independent screening of titles, abstracts, and full-text records and performed risk-of-bias assessment; OM: primary responsibility for the final content; and all authors: read and approved the final manuscript.

## Data availability

Data described in the manuscript are available from the corresponding author on reasonable request.

## Declaration of Generative AI and AI-assisted technologies in the writing process

During the preparation of this work, the authors used Microsoft Copilot to improve readability, sentence structure, and grammar. After using this tool, the authors critically reviewed and edited the content as needed and take full responsibility for the content of the final publication.

## Funding

ASB is supported by a Doctoral Research Scholarship administered by the School of Sport, Exercise, and Health Sciences and the Doctoral College at Loughborough University, United Kingdom. No external funding was received to support the conduct of this systematic review and meta-analysis.

## Conflict of interest

OM has received related research funding from Arla Foods Ingredients Group P/S (AFI), Denmark, which supports doctoral research of ASB. AFI did not provide any funding for, or input into, the conduct of this systematic review and meta-analysis. YT declares no known competing financial interests or personal relationships that could have influenced the work reported in this paper.
