# Supplementary material for: Impact of Milk Fat Globule Membrane and Its Associated Polar Lipids on Markers of Cardiometabolic Disease and Cognitive Function: A Systematic Review and Meta-Analysis of Randomized Controlled Trials in Adults
Source: Adv Nutr. 2026 Jul 25;17(9):100694. doi: 10.1016/j.advnut.2026.100694 (PMC13470189; doi:10.1016/j.advnut.2026.100694)
Supplement: Supplementary file 1 [file mmc1.docx]

**Impact of Milk Fat Globule Membrane and its Associated Polar Lipids on Markers of Cardiometabolic Disease and Cognitive Function: A Systematic Review and Meta-Analysis of Randomized Controlled Trials in Adults**

Borkar et al.

Online supplemental material

**Supplemental Table 1**. Database searches

**Database:** PubMed

**Date:** 23 June 2025

**Results:** 261

| Search number | Query | Results |
| --- | --- | --- |
| 24 | #22 NOT #23 | 261 |
| 23 | infant*[Title] OR children[Title] OR infancy[Title] OR neonat*[Title] OR calve*[Title] OR pediatric[Title]OR review[Title] OR "Vitamin D"[Title] OR vitamin[Title] OR cholecalciferol[Title] OR lactoferrin[Title] OR calcium[Title] OR fortified[Title] OR omega-3[Title] OR hydroxyvitamin D[Title] OR mediterranean[Title] OR DASH[Title] OR ketogenic[Title] OR nordic[Title] OR "meta analysis"[Title] OR mice[Title] OR rat[Title] OR rats[Title] OR calf[Title] | 3,656,169 |
| 22 | #9 OR #11 OR #13 OR #15 OR #17 OR #19 OR #21 | 332 |
| 21 | #7 AND #20 | 24 |
| 20 | (gut microbiota[Title/Abstract] OR gut microbiome[Title/Abstract] OR gut bacteria[Title/Abstract] OR gut flora[Title/Abstract] OR gastrointestinal microbiota[Title/Abstract] OR gastrointestinal microbiome[Title/Abstract] OR intestinal microbiota[Title/Abstract] OR intestinal microbiome[Title/Abstract] OR colonic microbiota[Title/Abstract] OR colonic microbiome[Title/Abstract] OR microflora[Title/Abstract] OR gut dysbiosis[Title/Abstract] OR dysbiosis[Title/Abstract] OR gut-derived bacteria[Title/Abstract] OR gut-derived microbes[Title/Abstract] OR microbiota composition[Title/Abstract] OR bacterial composition[Title/Abstract] OR fecal microbiota[Title/Abstract] OR faecal microbiota[Title/Abstract] OR fecal bacteria[Title/Abstract] OR faecal bacteria[Title/Abstract] OR short-chain fatty acids[Title/Abstract] OR short chain fatty acids[Title/Abstract] OR SCFA[Title/Abstract] OR acetate[Title/Abstract] OR propionate[Title/Abstract] OR butyrate[Title/Abstract] OR volatile fatty acids[Title/Abstract] OR microbial metabolites[Title/Abstract] OR gut-derived metabolites[Title/Abstract] OR gut derived metabolites[Title/Abstract] OR microbiota-derived metabolites[Title/Abstract] OR microbiota derived metabolites[Title/Abstract] OR gut metabolites[Title/Abstract] OR gut-derived compounds[Title/Abstract] OR gut derived compounds[Title/Abstract] OR microbiota-produced metabolites[Title/Abstract] OR microbiota produced metabolites[Title/Abstract] OR postbiotics[Title/Abstract] OR trimethylamine N-oxide[Title/Abstract] OR trimethylamine-N-oxide[Title/Abstract] OR trimethylamine oxide[Title/Abstract] OR trimethylammonium oxide[Title/Abstract] OR TMAO[Title/Abstract] OR gut barrier[Title/Abstract] OR intestinal barrier[Title/Abstract] OR gastrointestinal barrier[Title/Abstract] OR enteric barrier[Title/Abstract] OR intestinal epithelial barrier[Title/Abstract] OR epithelial barrier integrity[Title/Abstract] OR leaky gut[Title/Abstract] OR gut permeability[Title/Abstract] OR intestinal permeability[Title/Abstract] OR barrier function[Title/Abstract] OR barrier dysfunction[Title/Abstract] OR barrier integrity[Title/Abstract] OR barrier permeability[Title/Abstract]) OR Gastrointestinal Microbiome[MeSh Terms] OR Intestinal Barrier Function[MeSh Terms] OR Fatty Acids, Volatile[MeSh Terms] OR trimethyloxamine [Supplementary Concept] | 473,145 |
| 19 | #7 AND #18 | 12 |
| 18 | (vascular function[Title/Abstract] OR vascular stiffness[Title/Abstract] OR arterial stiffness[Title/Abstract] OR aortic stiffness[Title/Abstract] OR pulse wave velocity[Title/Abstract] OR PWV[Title/Abstract] OR pulse wave analysis[Title/Abstract] OR PWA[Title/Abstract] OR augmentation index[Title/Abstract] OR augmentation pressure[Title/Abstract] OR AIx[Title/Abstract] OR AIx@HR75[Title/Abstract] OR Plethysmograph[Title/Abstract] OR Laser-Doppler Flowmetry[Title/Abstract] OR Laser-Doppler Imaging[Title/Abstract] OR flow-mediated dilation[Title/Abstract] OR flow mediated dilatation[Title/Abstract] OR flow-mediated dilation[Title/Abstract] OR flow-mediated dilatation[Title/Abstract] OR FMD[Title/Abstract] OR arterial pressure[Title/Abstract] OR mean arterial pressure[Title/Abstract]) OR Vascular Stiffness[MeSh Terms] OR Blood Flow Velocity[MeSh Terms] OR Arterial Pressure[MeSh Terms] OR Pulse Wave Analysis[MeSh Terms] OR Plethysmography[MeSh Terms] OR Laser-Doppler Flowmetry[MeSh Terms] | 200,971 |
| 17 | #7 AND #16 | 50 |
| 16 | ((inflammation[Title/Abstract] OR inflammatory[Title/Abstract] OR anti-inflammatory[Title/Abstract]) AND (marker[Title/Abstract] OR biomarker[Title/Abstract])) OR interleukin[Title/Abstract] OR interleukin-6[Title/Abstract] OR interleukin 6[Title/Abstract] OR IL6[Title/Abstract] OR IL-6[Title/Abstract] OR tumour necrosis factor alpha[Title/Abstract] OR TNF-α[Title/Abstract] OR TNF-alpha[Title/Abstract] OR TNFα[Title/Abstract] OR TNF alpha[Title/Abstract] OR C-reactive protein[Title/Abstract] OR CRP[Title/Abstract] OR serum amyloid A[Title/Abstract] OR SAA[Title/Abstract] OR lipopolysaccharide[Title/Abstract] OR LPS[Title/Abstract] OR LBP[Title/Abstract] OR Cytokines[MeSh Terms] OR C-Reactive Protein[MeSh Terms] OR Interleukins[MeSh Terms] OR Serum Amyloid A Protein[MeSh Terms] OR Lipopolysaccharide-Binding Protein[Supplementary Concept] | 1,261,053 |
| 15 | #7 AND #14 | 103 |
| 14 | insulin resistance[Title/Abstract] OR insulin sensitivity[Title/Abstract] OR glucose[Title/Abstract] OR Homeostatic Model Assessment of Insulin Resistance[Title/Abstract] OR HOMA-IR[Title/Abstract] OR glycemic[Title/Abstract] OR glycaemic[Title/Abstract] OR HbA1c[Title/Abstract] OR glycated hemoglobin[Title/Abstract] OR glycated haemoglobin[Title/Abstract] OR Insulin Resistance[MeSh Terms] OR Blood Glucose[MeSh Terms] OR Hyperglycemia[MeSh Terms] OR Glycated Hemoglobin[MeSh Terms] OR Glycemic Control[MeSh Terms] | 818,558 |
| 13 | #7 AND #12 | 14 |
| 12 | "cognitive function"[Title/Abstract] OR "cognition"[Title/Abstract] OR "cognitive decline"[Title/Abstract] OR cognitive[Title/Abstract] OR cognitive performance[Title/Abstract] OR "mental performance"[Title/Abstract] OR "memory"[Title/Abstract] OR "working memory"[Title/Abstract] OR "executive function"[Title/Abstract] OR "cognitive flexibility"[Title/Abstract] OR "processing speed"[Title/Abstract] OR "visuospatial"[Title/Abstract] OR "spatial recognition"[Title/Abstract] OR "learning"[Title/Abstract] OR "attention"[Title/Abstract] OR "verbal fluency"[Title/Abstract] OR "cognitive ability"[Title/Abstract] OR "semantic fluency"[Title/Abstract] OR "phonemic fluency"[Title/Abstract] OR "decision making"[Title/Abstract] OR "reasoning"[Title/Abstract] OR "language function"[Title/Abstract] OR "problem solving"[Title/Abstract] OR "Neurocognitive Test Battery"[Title/Abstract] OR "NTB"[Title/Abstract] OR "Cognitive Drug Research"[Title/Abstract] OR "CDR"[Title/Abstract] OR "Cambridge Neuropsychological Test Automated Battery"[Title/Abstract] OR "CANTAB"[Title/Abstract] OR "Mini Mental State Examination"[Title/Abstract] OR "MMSE"[Title/Abstract] OR "Repeatable Battery for the Assessment of Neuropsychological Status"[Title/Abstract] OR "RBANS"[Title/Abstract] OR "Addenbrooke’s Cognitive Examination"[Title/Abstract] OR "ACE"[Title/Abstract] OR "Montreal Cognitive Assessment"[Title/Abstract] OR "MoCA"[Title/Abstract] OR "BDNF"[Title/Abstract] OR "brain-derived neurotrophic factor" amyloid beta[Title/Abstract] OR "amyloid-beta"[Title/Abstract] OR "Amyloid β"[Title/Abstract] OR "Amyloid-β"[Title/Abstract] OR "Aβ"[Title/Abstract] OR "Abeta"[Title/Abstract] OR "beta amyloid"[Title/Abstract] OR "beta-amyloid"[Title/Abstract] OR "Aβ42/Aβ40 ratio"[Title/Abstract] OR "Aβ 42/40 ratio"[Title/Abstract] OR "amyloid beta 42/40 ratio"[Title/Abstract] OR "beta-amyloid 42/40 ratio"[Title/Abstract] OR "phosphorylated tau"[Title/Abstract] OR "p-tau"[Title/Abstract] OR "ptau"[Title/Abstract] OR "Phospho-tau"[Title/Abstract] OR "Cognition"[MeSh Terms] OR "Neuropsychological Tests"[MeSh Terms] OR "Thinking"[MeSh Terms] OR "Learning"[MeSh Terms] OR "Executive Function"[MeSh Terms] OR "Amyloid beta-Peptides"[MeSh Terms] OR "Brain-Derived Neurotrophic Factor"[MeSh Terms] OR "tau Proteins"[MeSh Terms] | 1,120,568 |
| 11 | #7 AND #10 | 83 |
| 10 | hypertension[Title/Abstract] OR blood pressure[Title/Abstract] OR systolic[Title/Abstract] OR diastolic[Title/Abstract] OR Hypertension[MeSh Terms] OR Blood Pressure[MeSh Terms] OR Blood Pressure Determination[MeSh Terms] | 1,045,691 |
| 9 | #7 AND #8 | 288 |
| 8 | "hypertriglycerid*"[Title/Abstract] OR "triglycerides"[Title/Abstract] OR "triacylglycerol"[Title/Abstract] OR "TG"[Title/Abstract] OR "TAG"[Title/Abstract] OR "lipid profile"[Title/Abstract] OR "lipid levels"[Title/Abstract] OR "lipid metabolism"[Title/Abstract] OR "lipid parameters"[Title/Abstract] OR "serum lipid*"[Title/Abstract] OR "plasma lipid*"[Title/Abstract] OR Triglycerides[MeSh Terms] OR Lipid Metabolism[MeSh Terms] OR chol[Title/Abstract] OR cholesterol[Title/Abstract] OR HDL[Title/Abstract] OR LDL[Title/Abstract] OR VLDL[Title/Abstract] OR lipoproteins[Title/Abstract] OR apolipoproteins[Title/Abstract] OR apob[Title/Abstract] OR apoa[Title/Abstract] OR Cholesterol[MeSh Terms] OR Lipoproteins[MeSh Terms] OR Apolipoproteins[MeSh Terms] OR Cholesterol, LDL[MeSh Terms] OR Cholesterol, HDL[MeSh Terms] OR Cholesterol, VLDL[MeSh Terms] OR hyperlipidemia[Title/Abstract] OR hyperlipidaemia[Title/Abstract] OR hypercholesterolemia[Title/Abstract] OR hypertriglyceridemia[Title/Abstract] OR dyslipidemia[Title/Abstract] OR dyslipidaemia[Title/Abstract] OR Hyperlipidemias[MeSh Terms] OR Hypertriglyceridemia[MeSh Terms] OR Hypercholesterolemia[MeSh Terms] | 782,192 |
| 7 | #3 AND #4 AND #5 AND #6 | 484 |
| 6 | "randomized controlled trial"[Title/Abstract] OR "randomised controlled trial"[Title/Abstract] OR RCT[Title/Abstract] OR "controlled clinical trial"[Title/Abstract] OR randomized[Title/Abstract] OR randomised[Title/Abstract] OR placebo[Title/Abstract] OR intervention[Title/Abstract] OR randomly[Title/Abstract] OR experimental[Title/Abstract] OR control[Title/Abstract] OR parallel[Title/Abstract] OR crossover[Title/Abstract] OR postprandial[Title/Abstract] OR Controlled Clinical Trial[Publication Type] | 6,631,266 |
| 5 | "adult"[Title/Abstract] OR "adults"[Title/Abstract] OR "elderly"[Title/Abstract] OR "middle-aged"[Title/Abstract] OR "senior"[Title/Abstract] OR "seniors"[Title/Abstract] OR "aging"[Title/Abstract] OR "geriatric"[Title/Abstract] OR "ageing"[Title/Abstract] OR "aged"[Title/Abstract] OR "older"[Title/Abstract] OR "geront"[Title/Abstract] OR "healthy volunteers"[Title/Abstract] OR "men"[Title/Abstract] OR "women"[Title/Abstract] OR "postmenopausal"[Title/Abstract] OR "adult"[MeSH Terms] | 10,491,704 |
| 4 | "bovine"[Title/Abstract] OR "dairy"[Title/Abstract] OR "cow"[Title/Abstract] OR "buttermilk"[Title/Abstract] | 314,426 |
| 3 | #1 OR #2 | 622,401 |
| 2 | ("milk"[Title/Abstract] AND ("phospholipids"[Title/Abstract] OR "phosphatidylcholine"[Title/Abstract] OR "phosphatidylethanolamine"[Title/Abstract] OR "phosphatidylserine"[Title/Abstract] OR "phosphatidylinositol"[Title/Abstract] OR "sphingomyelin"[Title/Abstract] OR "sphingolipids"[Title/Abstract] OR "gangliosides"[Title/Abstract] OR "lecithin"[Title/Abstract] OR "polar lipids"[Title/Abstract])) OR "whey protein phospholipid concentrate"[Title/Abstract] OR Phospholipids [Mesh terms] OR Glycolipids[MeSh Terms] OR membrane lipids[MeSh Terms] | 447,717 |
| 1 | "milk fat globule membrane"[Title/Abstract] OR "milk fat globule membranes"[Title/Abstract] OR "milk fat globules"[Title/Abstract] OR "milk fat globule"[Title/Abstract] OR "MFGM"[Title/Abstract] OR "glycolipid*"[Title/Abstract] OR "glycoprotein*"[Title/Abstract] OR "complex milk lipid"[Title/Abstract] OR "complex milk lipids"[Title/Abstract] OR "milk polar lipids"[Title/Abstract] OR "milk polar lipid"[Title/Abstract] OR "milk-derived lipids"[Title/Abstract] OR milk fat globule [Supplementary Concept] | 191,315 |

**Database:** Embase

**Date:** 23 June 2025

**Results:** 261

| No. | Query | Results |
| --- | --- | --- |
| #24 | #22 NOT #23 | 261 |
| #23 | 'infant*':ti OR 'children':ti OR 'infancy':ti OR 'neonat*':ti OR 'pediatric':ti OR 'calve*':ti OR 'review':ti OR 'vitamin d':ti OR 'vitamin':ti OR 'cholecalciferol':ti OR 'lactoferrin':ti OR 'calcium':ti OR 'fortified':ti OR 'omega-3':ti OR 'hydroxyvitamin d':ti OR 'mediterranean':ti OR 'dash':ti OR 'ketogenic':ti OR 'nordic':ti OR 'meta analysis':ti OR 'mice':ti OR 'rat':ti OR 'rats':ti OR 'calf':ti | 4514529 |
| #22 | #9 OR #11 OR #13 OR #15 OR #17 OR #19 OR #21 | 217 |
| #21 | #7 AND #20 | 9 |
| #20 | ((microbiot* OR microbiom* OR microflora OR dysbiosis OR 'microbiota composition' OR 'bacterial composition') NEAR/3 (gut OR intestinal OR gastrointestinal OR colonic OR fecal OR faecal OR enteric)) OR 'short-chain fatty acid*':ti,ab,kw OR scfa*:ti,ab,kw OR acetate:ti,ab,kw OR propionate:ti,ab,kw OR butyrate:ti,ab,kw OR 'volatile fatty acid*':ti,ab,kw OR 'microbial metabolite*':ti,ab,kw OR 'gut metabolite*':ti,ab,kw OR 'gut-derived compound*':ti,ab,kw OR 'microbiota-derived metabolite*':ti,ab,kw OR postbiotic*:ti,ab,kw OR tmao*:ti,ab,kw OR 'trimethylamine oxide':ti,ab,kw OR 'trimethylamine-n-oxide':ti,ab,kw OR 'trimethylamine n-oxide':ti,ab,kw OR 'trimethylammonium oxide':ti,ab,kw OR gut:ti,ab,kw OR 'barrier function':ti,ab,kw OR 'barrier dysfunction':ti,ab,kw OR 'barrier integrity':ti,ab,kw OR 'barrier permeability':ti,ab,kw OR 'leaky gut':ti,ab,kw OR 'epithelial barrier integrity':ti,ab,kw OR ((barrier OR permeability) NEAR/3 (gut OR intestinal OR gastrointestinal OR enteric OR epithelial)) OR 'intestine flora'/exp OR 'intestinal barrier function'/exp OR 'intestine mucosa permeability'/exp OR 'volatile fatty acid'/exp | 615478 |
| #19 | #7 AND #18 | 3 |
| #18 | (('vascular' OR 'arterial' OR 'aortic') NEAR/3 (function* OR stiffness)) OR 'pulse wave velocity':ti,ab,kw OR pwv:ti,ab,kw OR 'pulse wave analysis':ti,ab,kw OR pwa:ti,ab,kw OR 'augmentation index':ti,ab,kw OR aix:ti,ab,kw OR 'augmentation pressure':ti,ab,kw OR 'aix@hr75':ti,ab,kw OR 'plethysmograph':ti,ab,kw OR 'laser-doppler flowmetry':ti,ab,kw OR 'laser-doppler imaging':ti,ab,kw OR 'flow mediated dilatation':ti,ab,kw OR 'flow mediated dilation':ti,ab,kw OR fmd:ti,ab,kw OR 'arterial pressure':ti,ab,kw OR 'mean arterial pressure':ti,ab,kw OR 'arterial stiffness'/exp OR 'heart atrium pressure'/exp OR 'plethysmography'/exp OR 'pulse wave'/exp OR 'laser doppler flowmetry'/exp | 279071 |
| #17 | #7 AND #16 | 36 |
| #16 | (inflammat*:ti,ab,kw OR 'anti-inflammat*':ti,ab,kw) AND (marker*:ti,ab,kw OR biomarker*:ti,ab,kw) OR interleukin*:ti,ab,kw OR 'interleukin-6':ti,ab,kw OR il6:ti,ab,kw OR 'il-6':ti,ab,kw OR 'tumor necrosis factor alpha':ti,ab,kw OR 'tnf alpha':ti,ab,kw OR crp:ti,ab,kw OR 'c-reactive protein':ti,ab,kw OR 'serum amyloid a':ti,ab,kw OR saa:ti,ab,kw OR lipopolysaccharide:ti,ab,kw OR lps:ti,ab,kw OR lbp:ti,ab,kw OR 'lipopolysaccharide-binding protein':ti,ab,kw OR 'inflammation'/exp OR 'c reactive protein'/exp OR 'interleukin 6'/exp OR 'tumor necrosis factor'/exp OR 'cytokine'/exp OR 'interleukin'/exp OR 'serum amyloid a'/exp | 6699534 |
| #15 | #7 AND #14 | 12 |
| #14 | 'insulin resistance':ti,ab,kw OR 'insulin sensitivity':ti,ab,kw OR glucose:ti,ab,kw OR 'homeostatic model assessment of insulin resistance':ti,ab,kw OR 'homa ir':ti,ab,kw OR glycemic:ti,ab,kw OR glycaemic:ti,ab,kw OR hba1c:ti,ab,kw OR 'glycated hemoglobin':ti,ab,kw OR 'glycated haemoglobin':ti,ab,kw OR 'insulin resistance'/exp OR 'glucose blood level'/exp OR 'glycated hemoglobin'/exp OR 'glycemic control'/exp | 1223185 |
| #13 | #7 AND #12 | 11 |
| #12 | (cognit* NEAR/3 (function* OR declin* OR reduc* OR impair* OR improv* OR deficit* OR progress* OR perform* OR abilit* OR marker* OR biomarker*)) OR cognition:ti,ab,kw OR 'mental perform*':ti,ab,kw OR memory:ti,ab,kw OR 'working memory':ti,ab,kw OR 'executive function*':ti,ab,kw OR 'cognitive flexibility':ti,ab,kw OR 'processing speed':ti,ab,kw OR 'speed of processing':ti,ab,kw OR visuospatial:ti,ab,kw OR 'spatial recognition':ti,ab,kw OR learning:ti,ab,kw OR attention:ti,ab,kw OR 'verbal fluency':ti,ab,kw OR 'semantic fluency':ti,ab,kw OR 'phonemic fluency':ti,ab,kw OR 'decision making':ti,ab,kw OR reasoning:ti,ab,kw OR 'language function':ti,ab,kw OR 'problem solving':ti,ab,kw OR 'neurocognitive test battery':ti,ab,kw OR ntb:ti,ab,kw OR 'cognitive drug research':ti,ab,kw OR cdr:ti,ab,kw OR 'cambridge neuropsychological test automated battery':ti,ab,kw OR cantab:ti,ab,kw OR 'mini mental state examination':ti,ab,kw OR mmse:ti,ab,kw OR 'repeatable battery for the assessment of neuropsychological status':ti,ab,kw OR rbans:ti,ab,kw OR 'addenbrookes cognitive examination':ti,ab,kw OR ace:ti,ab,kw OR 'montreal cognitive assessment':ti,ab,kw OR moca:ti,ab,kw OR bdnf:ti,ab,kw OR 'brain derived neurotrophic factor':ti,ab,kw OR 'amyloid beta':ti,ab,kw OR 'amyloid-beta':ti,ab,kw OR 'amyloid β':ti,ab,kw OR 'amyloid-β':ti,ab,kw OR aβ:ti,ab,kw OR abeta:ti,ab,kw OR 'beta amyloid':ti,ab,kw OR 'beta-amyloid':ti,ab,kw OR 'aβ42/aβ40 ratio':ti,ab,kw OR 'aβ 42/40 ratio':ti,ab,kw OR 'amyloid beta 42/40 ratio':ti,ab,kw OR 'beta-amyloid 42/40 ratio':ti,ab,kw OR 'phosphorylated tau':ti,ab,kw OR 'phospho-tau':ti,ab,kw OR ptau:ti,ab,kw OR 'p-tau':ti,ab,kw OR 'cognition'/exp OR 'executive function'/exp OR 'learning'/exp OR 'thinking'/exp OR 'spatial orientation'/exp OR 'brain derived neurotrophic factor'/exp OR 'tau protein'/exp OR 'amyloid protein'/exp | 5060766 |
| #11 | #7 AND #10 | 16 |
| #10 | hypertension:ti,ab,kw OR 'blood pressure':ti,ab,kw OR systolic:ti,ab,kw OR diastolic:ti,ab,kw OR 'hypertension'/exp OR 'blood pressure'/exp | 2153800 |
| #9 | #7 AND #8 | 169 |
| #8 | hypertriglycerid:ti,ab,kw OR triglycerides:ti,ab,kw OR triacylglycerol:ti,ab,kw OR tg:ti,ab,kw OR 'lipid profile':ti,ab,kw OR 'lipid levels':ti,ab,kw OR 'lipid metabolism':ti,ab,kw OR 'lipid parameters':ti,ab,kw OR 'plasma lipid*':ti,ab,kw OR 'serum lipid*':ti,ab,kw OR 'triglyceride'/exp OR 'lipid'/exp OR 'lipid metabolism'/exp OR chol:ti,ab,kw OR cholesterol:ti,ab,kw OR hdl*:ti,ab,kw OR ldl*:ti,ab,kw OR vldl*:ti,ab,kw OR lipoproteins:ti,ab,kw OR apolipoproteins:ti,ab,kw OR apob:ti,ab,kw OR apoa:ti,ab,kw OR 'cholesterol'/exp OR 'lipoprotein'/exp OR hyperlipidemi*:ti,ab,kw OR hyperlipidaemi*:ti,ab,kw OR hypercholesterolemi*:ti,ab,kw OR hypertriglyceridemi*:ti,ab,kw OR dyslipidemi*:ti,ab,kw OR dyslipidaemi*:ti,ab,kw OR 'hyperlipidemia'/exp | 2796566 |
| #7 | #3 AND #4 AND #5 AND #6 | 274 |
| #6 | 'randomized controlled trial':ti,ab,kw OR 'randomised controlled trial':ti,ab,kw OR rct:ti,ab,kw OR 'controlled clinical trial':ti,ab,kw OR randomized:ti,ab,kw OR randomised:ti,ab,kw OR placebo:ti,ab,kw OR intervention:ti,ab,kw OR randomly:ti,ab,kw OR experimental:ti,ab,kw OR control:ti,ab,kw OR parallel:ti,ab,kw OR crossover:ti,ab,kw OR postprandial:ti,ab,kw OR 'double blind':ti,ab,kw OR blinded:ti,ab,kw | 8696873 |
| #5 | adult:ti,ab,kw OR adults:ti,ab,kw OR elderly:ti,ab,kw OR 'middle aged':ti,ab,kw OR senior:ti,ab,kw OR seniors:ti,ab,kw OR aging:ti,ab,kw OR geriatric:ti,ab,kw OR ageing:ti,ab,kw OR aged:ti,ab,kw OR older:ti,ab,kw OR geront:ti,ab,kw OR 'healthy volunteers':ti,ab,kw OR men:ti,ab,kw OR women:ti,ab,kw OR postmenopausal:ti,ab,kw OR 'adult'/exp | 15171286 |
| #4 | bovine:ti,ab,kw OR dairy:ti,ab,kw OR cow:ti,ab,kw OR buttermilk:ti,ab,kw | 355162 |
| #3 | #1 OR #2 | 706119 |
| #2 | milk:ti,ab,kw AND (phospholipid*:ti,ab,kw OR phosphatidylcholine:ti,ab,kw OR phosphatidylethanolamine:ti,ab,kw OR phosphatidylserine:ti,ab,kw OR phosphatidylinositol:ti,ab,kw OR sphingomyelin:ti,ab,kw OR sphingolipid*:ti,ab,kw OR ganglioside*:ti,ab,kw OR lecithin:ti,ab,kw OR 'polar lipid*':ti,ab,kw) OR 'whey protein phospholipid concentrate':ti,ab,kw OR 'bioactive milk lipid*':ti,ab,kw OR 'bioactive milk fat*':ti,ab,kw OR ('milk lipid*':ti,ab,kw AND bioactive:ti,ab,kw) OR (buttermilk:ti,ab,kw AND (phospholipid*:ti,ab,kw OR sphingolipid*:ti,ab,kw OR 'polar lipid':ti,ab,kw)) OR 'phospholipid'/exp OR 'sphingolipid'/exp OR 'membrane lipid'/exp | 388925 |
| #1 | 'milk fat globule membrane'/exp OR 'milk fat globule membrane' OR 'milk fat globules' OR 'milk fat globule'/exp OR 'milk fat globule' OR mfgm OR 'milk lipid complex' OR glycolipid* OR glycoprotein* OR 'complex milk lipid':ti,ab,kw | 338701 |

**Database:** Cochrane CENTRAL

**Date:** 23 June 2025

**Results:** 191

ID	Search
#1	((milk NEXT fat NEXT globule NEXT (membrane OR membrane-enriched)) OR "milk fat globules" OR "milk fat globule" OR MFGM OR "milk lipid complex" OR glycolipid* OR glycoprotein* OR "complex milk lipid" OR "complex milk lipids"):ti,ab,kw
#2	((milk NEXT (phosphatidylcholine OR phosphatidylethanolamine OR phosphatidylserine OR phosphatidylinositol OR sphingomyelin OR sphingolipid* OR ganglioside* OR lecithin OR polar NEXT lipid*)) OR "whey protein phospholipid concentrate" OR milk-PL OR bioactive NEXT milk NEXT lipid* OR "milk lipid concentrate" OR milk-derived NEXT lipid* OR milk phospholipids OR milk phospholipid OR milk-derived NEXT polar NEXT lipid* OR (milk NEXT lipid* AND bioactive) OR (buttermilk AND (phospholipid* OR sphingolipid* OR polar lipid))):ti,ab,kw
#3	MeSH descriptor: [Phospholipids] explode all trees
#4	MeSH descriptor: [Sphingolipids] explode all trees
#5	MeSH descriptor: [Membrane Lipids] explode all trees
#6	#1 OR #2 OR #3 OR #4 OR #5
#7	#1 OR #6
#8	(bovine OR dairy OR cow*):ti,ab,kw
#9	(adult OR adults OR elderly OR middle-aged OR senior OR seniors OR aging OR geriatric OR ageing OR aged OR older OR geront OR healthy volunteers OR men OR women):ti,ab,kw
#10	MeSH descriptor: [Adult] explode all trees
#11	#9 OR #10
#12	#7 AND #8
#13	("randomized controlled trial" OR "randomised controlled trial" OR RCT OR "controlled clinical trial" OR randomized OR randomised OR placebo OR intervention OR randomly OR experimental OR control OR parallel OR crossover OR postprandial):ti,ab,kw
#14	#12 AND #13
#15	(hypertriglycerid* OR triglyceride* OR triacylglycerol* OR TG* OR lipid profile* OR lipid level* OR lipid metabolism OR TAG):ti,ab,kw
#16	MeSH descriptor: [Triglycerides] explode all trees
#17	MeSH descriptor: [Lipids] explode all trees
#18	MeSH descriptor: [Lipid Metabolism] explode all trees
#19	#15 OR #16 OR #17 OR #18
#20	(cholesterol OR chol* OR HDL* OR LDL* OR VLDL* OR lipoprotein* OR apolipoprotein* OR apob OR apoa):ti,ab,kw
#21	MeSH descriptor: [Cholesterol] explode all trees
#22	MeSH descriptor: [Lipoproteins] explode all trees
#23	#20 OR #21 #22
#24	(hyperlipidaemi* OR hyperlipidemi* OR dyslipidemi* OR dyslipidaemi* OR hypercholesterol* OR hypertriglycerid* OR lipid-parameters):ti,ab,kw
#25	MeSH descriptor: [Hyperlipidemias] explode all trees
#26	#24 OR #25
#27	#14 AND (#19 OR #23 OR #26)
#28	(hypertension OR "blood pressure" OR systolic OR diastolic):ti,ab,kw
#29	MeSH descriptor: [Hypertension] explode all trees
#30	MeSH descriptor: [Blood Pressure] explode all trees
#31	#28 OR #29 OR #30
#32	#14 AND #31
#33	((cognit* NEAR/3 (function* OR declin* OR reduc* OR impair* OR improv* OR deficit* OR progress* OR perform* OR abilit* OR marker* OR biomarker*)) OR cognition OR mental NEXT perform* OR memory OR "working memory" OR executive NEXT function* OR "cognitive flexibility" OR "processing speed" OR "speed of processing" OR visuospatial OR "spatial recognition" OR learning OR attention OR "verbal fluency" OR "semantic fluency" OR "phonemic fluency" OR "decision making" OR reasoning OR "language function" OR "problem solving" OR "Neurocognitive Test Battery" OR NTB OR "Cognitive Drug Research" OR CDR OR "Cambridge Neuropsychological Test Automated Battery" OR CANTAB OR "Mini Mental State Examination" OR MMSE OR "Repeatable Battery for the Assessment of Neuropsychological Status" OR RBANS OR "Addenbrooke’s Cognitive Examination" OR ACE OR "Montreal Cognitive Assessment" OR MoCA OR BDNF OR "brain derived neurotrophic factor" OR "amyloid beta" OR "amyloid-beta" OR "Amyloid β" OR "Amyloid-β" OR Aβ OR Abeta OR "beta amyloid" OR "beta-amyloid" OR "Aβ42/Aβ40 ratio" OR "Aβ 42/40 ratio" OR "amyloid beta 42/40 ratio" OR "beta-amyloid 42/40 ratio" OR "phosphorylated tau" OR "phospho-tau" OR ptau OR "p-tau"):ti,ab,kw
#34	MeSH descriptor: [Executive Function] explode all trees
#35	MeSH descriptor: [Cognition] explode all trees
#36	MeSH descriptor: [Learning] explode all trees
#37	MeSH descriptor: [Thinking] explode all trees
#38	MeSH descriptor: [Spatial Navigation] explode all trees
#39	MeSH descriptor: [Brain-Derived Neurotrophic Factor] explode all trees
#40	MeSH descriptor: [Amyloidogenic Proteins] explode all trees
#41	#33 OR #34 OR #35 OR #36 OR #37 OR #38 OR #39 OR #40
#42	#14 AND #41
#43	("insulin resistance" OR "insulin sensitivity" OR glucose OR "Homeostatic Model Assessment of Insulin Resistance" OR HOMA-IR OR glycemic OR glycaemic OR HbA1c OR "glycated hemoglobin" OR "glycated haemoglobin"):ti,ab,kw
#44	MeSH descriptor: [Insulin Resistance] explode all trees
#45	MeSH descriptor: [Blood Glucose] explode all trees
#46	MeSH descriptor: [Glycated Hemoglobin] explode all trees
#47	MeSH descriptor: [Glycemic Control] explode all trees
#48	#43 OR #44 OR #45 OR #46 OR #47
#49	#14 AND #48
#50	(((inflammatory OR anti-inflammatory OR inflammation OR anti-inflammation) AND (markers OR marker OR biomarkers OR biomarker)) OR interleukin* OR "interleukin-6" OR "interleukin 6" OR IL6 OR IL-6 OR "tumor necrosis factor alpha" OR "tumour necrosis factor alpha" OR TNF-alpha OR TNF-α OR TNFα OR CRP OR "C-reactive protein" OR "serum amyloid A" OR SAA OR lipopolysaccharide OR LPS OR LBP OR "lipopolysaccharide-binding protein"):ti,ab,kw
#51	MeSH descriptor: [Inflammation] explode all trees
#52	MeSH descriptor: [C-Reactive Protein] explode all trees
#53	MeSH descriptor: [Interleukin-6] explode all trees
#54	MeSH descriptor: [Tumor Necrosis Factor-alpha] explode all trees
#55	MeSH descriptor: [Cytokines] explode all trees
#56	MeSH descriptor: [Interleukins] explode all trees
#57	MeSH descriptor: [Serum Amyloid A Protein] explode all trees
#58	#50 OR #51 OR #52 OR #53 OR #54 OR #55 OR #56 OR #57
#59	#14 AND #58
#60	((("vascular" OR "arterial" OR "aortic") NEAR/3 (function* OR stiffness)) OR "pulse wave velocity" OR PWV OR "pulse wave analysis" OR PWA OR "augmentation index" OR AIx OR "augmentation pressure" OR "AIx@HR75" OR "Plethysmograph" OR "Laser-Doppler Flowmetry" OR "Laser-Doppler Imaging" OR "flow mediated dilatation" OR "flow mediated dilation" OR "flow-mediated dilation" OR "flow-mediated dilatation" OR FMD OR "arterial pressure" OR "mean arterial pressure"):ti,ab,kw
#61	MeSH descriptor: [undefined] explode all trees
#62	MeSH descriptor: [Atrial Pressure] explode all trees
#63	MeSH descriptor: [Atrial Function] explode all trees
#64	MeSH descriptor: [Plethysmography] explode all trees
#65	MeSH descriptor: [Pulse Wave Analysis] explode all trees
#66	MeSH descriptor: [Laser-Doppler Flowmetry] explode all trees
#67	#60 OR #61 OR #62 OR #63 OR #64 OR #65 OR #66
#68	#14 AND #67
#69	(((microbiot* OR microbiom* OR microflora OR dysbiosis OR "microbiota composition" OR "bacterial composition") NEAR/3 (gut OR intestinal OR gastrointestinal OR colonic OR fecal OR faecal OR enteric)) OR ((short-chain NEXT fatty NEXT acid* OR SCFA* OR acetate OR propionate OR butyrate OR volatile NEXT fatty NEXT acid*) OR ((microbial NEXT metabolite* OR gut NEXT metabolite* OR gut-derived NEXT compound* OR microbiota-derived NEXT metabolite* OR postbiotic*))) OR (TMAO* OR "trimethylamine oxide" OR "trimethylamine-N-oxide" OR "trimethylamine N-oxide" OR "trimethylammonium oxide") OR (("barrier function" OR "barrier dysfunction" OR "barrier integrity" OR "barrier permeability" OR ("leaky gut" OR "epithelial barrier integrity")) OR ((barrier OR permeability) NEAR/3 (gut OR intestinal OR gastrointestinal OR enteric OR epithelial)))):ti,ab,kw
#70	MeSH descriptor: [Gastrointestinal Microbiome] explode all trees
#71	MeSH descriptor: [Intestinal Barrier Function] explode all trees
#72	MeSH descriptor: [Fatty Acids, Volatile] explode all trees
#73	#69 OR #70 OR #71 OR #72
#74	#14 AND #73
#75	#27 OR #32 OR #42 OR #49 OR #59 OR #68 OR #74
#76	(infant* OR children OR infancy OR neonat* OR calve* OR review OR "Vitamin D" OR vitamin OR cholecalciferol OR lactoferrin OR calcium OR fortified OR omega-3 OR hydroxyvitamin D OR mediterranean OR DASH OR ketogenic OR nordic):ti
#77	#75 NOT #76

**Supplemental Table 2.** Data extraction form template

| **Study details** | | | | | | | | | | | |
| --- | --- | --- | --- | --- | --- | --- | --- | --- | --- | --- | --- |
| Initials of extractor | First author, year (Country) | | Title of paper | | Classification (Food-based/Supplement- based/mixed/unclear) | | | Sub-classification | | | Comparison |
| **Participant characteristics and health status** | | | | | | | | | | | |
| Health status | Age (mean or range) | | BMI (mean or range) | | Sex (M or M+F) | | | No. of participants who completed the study (% M/F) | | | Randomization process |
| **Study design: Intervention and comparator** | | | | | | | | | | | |
| Study design | No. of study arms | | | Type of intervention (Controlled/semi-controlled) | | | Duration of intervention (d) | | | Duration of washout (d) (Requirements) | |
| Intervention (Description and quantity consumed) | | Comparator (Description and quantity consumed) | | | Intake unit | Intake unit - other specify | | Controlled factors between treatments (relevant study arms only) | | | Method of dietary assessment |
| Mean dietary intake at baseline or run-in reported (Y/N) | | Relevant outcomes | | Duration of fast | | Methods of assessment (all relevant outcomes) | | | Primary outcome | | Power calculation details |
| Text summarising statistical approach | | Adjusted values? (Y/N) | | Confounders (list all) - specify if these vary between outcomes | | | Body mass similar between relevant study arms post-intervention (Y/N) | | | Text summarising all relevant outcomes | |
| **Outcome measures and results** | | | | | | | | | | | |
| Comparison | | Outcome | | | Units | | | Analytical sample (*n*) | | | |
| Mean values at baseline (+/- SD); or mean % prevalence at baseline (95% CIs) | | | Mean values post-intervention (± SD); or mean % prevalence post-intervention (95% CIs) | | | | Change in mean value baseline to post-intervention (± SE/SD) or change in % prevalence from baseline to post-intervention (95% CI) | | | | P-value |
| **Additional information** | | | | | | | | | | | |
| Sample size limitation (discussion) | | | Strengths and limitations | | | | Author's conclusion | | | Other notes (include relevant references) | |
| Notes (e.g. highlight missing information/issues with extraction etc) | | | Type of synthesis (i. narrative synthesis, ii. meta-analysis or iii. both) | | | | Industrial funding source (Yes/No/Not stated) | | | Authors’ declaration of interests | |

**Supplemental Table 3.** Overall risk of bias judgement assessment approach for randomized controlled trials using the Cochrane Risk of Bias tool 2.0^1^

| **Overall risk of bias judgement** | **Criteria** |
| --- | --- |
| Low risk of bias | The study is judged to be at low risk of bias for all domains for this result |
| Moderate risk of bias (Some concerns) | The study is judged to raise some concerns in at least one domain for this result, but not to be at high risk of bias for any domain |
| High risk of bias | The study is judged to be at risk of bias in at least one domain for this result, or the study is judged to have some concerns for multiple domains in a way that substantially lowers confidence in the result |

^1^From Sterne et al 2019 [1].

**Supplemental Results**

**Study-specific exclusions at full stage screening**

Several fasting studies [2–7] appeared to meet inclusion criteria at the title and abstract screening stage but were excluded for pre-defined reasons. Weiland et al. [6] (Trial 2) was excluded because study arms were not energy-matched. In Severins et al. [7], only the two beverage arms (buttermilk vs. skim-milk) met the inclusion criteria; the remaining two arms involved multi-nutrient co-supplementation. Pokala et al. [5] was excluded because the comparator beverage was not dairy-based. Studies in which MFGM was delivered alongside co-interventions, such as multi-nutrient supplementation with or without exercise, were also excluded [2–4].

Postprandial studies by Beals et al. [8], Demmer et al. [9], and Rogers et al. [10] were excluded because high-fat palm oil-based control meals were compared with meals where part of the fat source was replaced with MFGM. Although energy and fat contents were matched, this design introduced potential confounding, as observed effects could reflect both MFGM addition and palm oil reduction. Postprandial outcomes from the VALOBAB-C study (Vors et al. [11]) were excluded from synthesis because acute responses were assessed during a chronic intervention period (i.e., acute-within-chronic design).

**Supplemental Table 4.** Results of overall, subgroup, and sensitivity meta-analyses for fasting cardiometabolic outcomes with sufficient data from randomized control trials assessing the impact of MFGM/MPL intake versus control^1^

|  |  |  |  | **Results** | | **Heterogeneity** | |
| --- | --- | --- | --- | --- | --- | --- | --- |
| **Outcome** | **Subgroup^2 3^** | **Number of studies** | **Participants (*n*)** | **Meta-analysis Pooled effect (WMD [95% CI])** | ***p*^4^** | ***I^2^* (%)** | |
| TC | Overall | 10 [6,7,11–18] | 600 | -0.18 [-0.30, -0.07] mmol/L |  | 0 | |
|  | *Baseline health status* |  |  |  | 0.71 | 0 | |
|  | Healthy | 6 [12–15,17,18] | 386 | -0.16 [-0.37, 0.04] mmol/L |  | 11 | |
|  | At-risk | 4 [6,7,11,16] | 214 | -0.20 [-0.45, 0.04] mmol/L |  | 0 | |
|  | *Intervention duration* |  |  |  | 0.39 | 0 | |
|  | Short-term | 5 [11–15] | 182 | -0.13 [-0.36, 0.10] mmol/L |  | 11 | |
|  | Longer-term | 5 [6,7,16–18] | 418 | -0.22 [-0.42, -0.03] mmol/L |  | 0 | |
|  | *Intervention dosage* |  |  |  | 0.65 | 0 | |
|  | Low-dose | 3 [7,13,16] | 128 | -0.21 [-0.48, 0.06] mmol/L |  | 0 | |
|  | High-dose | 7 [6,11,12,14,15,17,18] | 472 | -0.16 [-0.36, 0.03] mmol/L |  | 21 | |
|  | *Overall subgroup differences* |  |  |  | 0.95 | 0 | |
|  | SA baseline imbalances | NA | NA | NA |  | NA | |
|  | SA (excluding Zajac et al.) | 9 [6,7,11–17] | 378 | -0.16 [-0.30, -0.02] mmol/L |  | 0 | |
| LDL-C | Overall | 10 [6,7,11–18] | 600 | -0.12 [-0.22, -0.03] mmol/L |  | 0 | |
|  | *Baseline health status* |  |  |  | 0.73 | 0 | |
|  | Healthy | 6 [12–15,17,18] | 386 | -0.11 [-0.24, 0.02] mmol/L |  | 0 | |
|  | At-risk | 4 [6,7,11,16] | 214 | -0.14 [-0.42, 0.13] mmol/L |  | 37 | |
|  | *Intervention duration* |  |  |  | 0.55 | 0 | |
|  | Short-term | 5 [11–15] | 182 | -0.09 [-0.26, 0.08] mmol/L |  | 15 | |
|  | Longer-term | 5 [6,7,16–18] | 418 | -0.15 [-0.32, 0.03] mmol/L |  | 7 | |
|  | *Intervention dosage* |  |  |  | 0.77 | 0 | |
|  | Low-dose | 3 [7,13,16] | 128 | -0.15 [-0.52, 0.22] mmol/L |  | 33 | |
|  | High-dose | 7 [6,11,12,14,15,17,18] | 472 | -0.12 [-0.24, 0.00] mmol/L |  | 0 | |
|  | *Overall subgroup differences* |  |  |  | 0.99 | 0 | |
|  | SA baseline imbalances | 8 [6,7,11,13–16,18] | 512 | -0.12 [-0.23, -0.00] mmol/L |  | 10 | |
|  | SA (excluding Zajac et al.) | 9 [6,7,11–17] | 378 | -0.11 [-0.24, 0.01] mmol/L |  | 17 | |
| HDL-C | Overall | 10 [6,7,11–18] | 600 | -0.01 [-0.06, 0.03] mmol/L |  | 13 | |
|  | *Baseline health status* |  |  |  | 0.02 | 82 | |
|  | Healthy | 6 [12–15,17,18] | 386 | -0.04 [-0.10, 0.02] mmol/L |  | 0 | |
|  | At-risk | 4 [6,7,11,16] | 214 | 0.02 [-0.03, 0.08] mmol/L |  | 0 | |
|  | *Intervention duration* |  |  |  | 0.90 | 0 | |
|  | Short-term | 5 [11–15] | 182 | -0.01 [-0.08, 0.07] mmol/L |  | 20 | |
|  | Longer-term | 5 [6,7,16–18] | 418 | -0.01 [-0.09, 0.06] mmol/L |  | 2 | |
|  | *Intervention dosage* |  |  |  | 0.44 | 0 | |
|  | Low-dose | 3 [7,13,16] | 128 | 0.01 [-0.04, 0.06] mmol/L |  | 0 | |
|  | High-dose | 7 [6,11,12,14,15,17,18] | 472 | -0.02 [-0.09, 0.06] mmol/L |  | 31 | |
|  | *Overall subgroup differences* |  |  |  | 0.25 | 24 | |
|  | SA baseline imbalances | 8 [6,7,11,13–16,18] | 512 | 0.00 [-0.04, 0.05] mmol/L |  | 17 | |
|  | SA (excluding Zajac et al.) | 9 [6,7,11–17] | 378 | -0.00 [-0.05, 0.05] mmol/L |  | 9 | |
| TC:HDL-C  ratio | Overall | 6 [6,7,11,13,14,16] | 258 | -0.17 [-0.33, -0.01] |  | 9 | |
|  | *Baseline health status* |  |  |  | 0.35 | 0 | |
|  | Healthy | 2 [13,14] | 44 | -0.11 [-0.66, 0.45] |  | 0 | |
|  | At-risk | 4 [6,7,11,16] | 214 | -0.20 [-0.47, 0.08] |  | 29 | |
|  | *Intervention duration* |  |  |  | 0.23 | 32 | |
|  | Short-term | 3 [11,13,14] | 102 | -0.28 [-0.79, 0.24] |  | 0 | |
|  | Longer-term | 3 [6,7,16] | 156 | -0.11 [-0.37, 0.14] |  | 0 | |
|  | *Intervention dosage* |  |  |  | 0.17 | 48 | |
|  | Low-dose | 3 [7,13,16] | 128 | -0.11 [-0.36, 0.14] |  | 0 | |
|  | High-dose | 3 [6,11,14] | 130 | -0.30 [-0.81, 0.22] |  | 0 | |
|  | *Overall subgroup differences* |  |  |  | 0.49 | 0 | |
|  | SA baseline imbalances | NA | NA | NA |  | NA | |
|  | SA (excluding Zajac et al.) | NA | NA | NA |  | NA | |
| Apo B | Overall | 5 [6,11,13,15,17] | 254 | -0.05 [-0.09, -0.01] g/L |  | 5 | |
|  | *Baseline health status* |  |  |  | 0.12 | 58 | |
|  | Healthy | 2 [12,17] | 88 | -0.03 [-0.04, -0.03] g/L |  | 0 | |
|  | At-risk | 3 [6,11,16] | 166 | -0.07 [-0.17, 0.03] g/L |  | 0 | |
|  | *Intervention duration* |  |  |  | 1.00 | 0 | |
|  | Short-term | 2 [11,12] | 106 | -0.05 [-0.31, 0.21] g/L |  | 30 | |
|  | Longer-term | 3 [6,16,17] | 148 | -0.05 [-0.20, 0.09] g/L |  | 20 | |
|  | *Overall subgroup differences* |  |  |  | 0.35 | 9 | |
| TG | Overall | 10 [6,7,11–18] | 600 | -0.09 [-0.20, 0.03] mmol/L |  | 43 | |
|  | *Baseline health status* |  |  |  | 0.01 | 83 | |
|  | Healthy | 6 [12–15,17,18] | 386 | -0.01 [-0.17, 0.14] mmol/L |  | 41 | |
|  | At-risk | 4 [6,7,11,16] | 214 | -0.20 [-0.34, -0.06] mmol/L |  | 0 | |
|  | *Intervention duration* |  |  |  | 0.71 | 0 | |
|  | Short-term | 5 [11–15] | 182 | -0.10 [-0.30, 0.09] mmol/L |  | 39 | |
|  | Longer-term | 5 [6,7,16–18] | 418 | -0.07 [-0.27, 0.14] mmol/L |  | 49 | |
|  | *Intervention dosage* |  |  |  | 0.26 | 21 | |
|  | Low-dose | 3 [7,13,16] | 128 | -0.14 [-0.31, 0.03] mmol/L |  | 0 | |
|  | High-dose | 7 [6,11,12,14,15,17,18] | 472 | -0.05 [-0.22, 0.11] mmol/L |  | 57 | |
|  | *Overall subgroup differences* |  |  |  | 0.15 | 38 | |
|  | SA baseline imbalances | 7 [6,11,13–16,18] | 464 | -0.11 [-0.24, 0.01] mmol/L |  | 15 | |
|  | SA (excluding Zajac et al.) | 9 [6,7,11–17] | 378 | -0.08 [-0.21, 0.05] mmol/L |  | 48 | |
| Apo A-I | Overall | 6 [6,7,11,12,16,17] | 302 | -0.01 [-0.05, 0.04] g/L |  | 18 | |
|  | *Baseline health status* |  |  |  | 0.01 | 84 | |
|  | Healthy | 2 [12,17] | 88 | -0.07 [-0.38, 0.24] g/L |  | 0 | |
|  | At-risk | 4 [6,7,11,16] | 214 | 0.00 [-0.04, 0.05] g/L |  | 5 | |
|  | *Intervention duration* |  |  |  | 0.50 | 0 | |
|  | Short-term | 2 [11,12] | 106 | -0.02 [-0.45, 0.40] g/L |  | 60 | |
|  | Longer-term | 4 [6,7,16,17] | 196 | 0.00 [-0.08, 0.09] g/L |  | 28 | |
|  | *Intervention dosage* |  |  |  | 0.61 | 0 | |
|  | Low-dose | 2 [7,16] | 94 | 0.01 [-0.42, 0.44] g/L |  | 56 | |
|  | High-dose | 4 [6,11,12,17] | 208 | -0.02 [-0.11, 0.08] g/L |  | 37 | |
|  | *Overall subgroup differences* |  |  |  | 0.21 | 30 | |
| Glucose | Overall | 5 [6,11,15–17] | 238 | -0.03 [-0.18, 0.12] mmol/L |  | 0 | |
|  | *Baseline health status* |  |  |  | 0.79 | 0 | |
|  | Healthy | 2 [15,17] | 72 | 0.00 [-1.82, 1.83] mmol/L |  | 0 | |
|  | At-risk | 3 [6,11,16] | 166 | -0.04 [-0.35, 0.27] mmol/L |  | 0 | |
|  | *Intervention duration* |  |  |  | 0.42 | 0 | |
|  | Short-term | 2 [11,15] | 90 | -0.00 [-0.49, 0.48] mmol/L |  | 0 | |
|  | Longer-term | 3 [6,16,17] | 148 | -0.10 [-0.56, 0.37] mmol/L |  | 33 | |
|  | *Overall subgroup differences* |  |  |  | 0.85 | 0 | |
| SBP | Overall | 5 [6,11,15,17,19] | 226 | -0.02 [-4.73, 4.69] mmHg |  | 3 | |
|  | *Baseline health status* |  |  |  | 0.13 | 56 | |
|  | Healthy | 3 [15,17,19] | 106 | -1.75 [-12.84, 9.34] mmHg |  | 16 | |
|  | At-risk | 2 [6,11] | 120 | 2.32 [-8.04, 12.67] mmHg |  | 0 | |
|  | *Intervention duration* |  |  |  | 0.76 | 0 | |
|  | Short-term | 3 [11,15,19] | 124 | 0.30 [-7.39, 7.98] mmHg |  | 6 | |
|  | Longer-term | 2 [6,17] | 102 | -1.43 [-68.07, 65.22] mmHg |  | 59 | |
|  | *Overall subgroup differences* |  |  |  | 0.35 | 10 | |
| DBP | Overall | 5 [6,11,15,17,19] | 226 | -1.05 [-3.78, 1.68] mmHg |  | 0 | |
|  | *Baseline health status* |  |  |  | 0.84 | 0 | |
|  | Healthy | 3 [15,17,19] | 106 | -0.65 [-5.29, 3.99] mmHg |  | 0 | |
|  | At-risk | 2 [6,11] | 120 | -1.16 [-30.20, 27.88] mmHg |  | 50 | |
|  | *Intervention duration* |  |  |  | < 0.0001 | 95 | |
|  | Short-term | 3 [11,15,19] | 124 | -2.14 [-5.00, 0.72] mmHg |  | 0 | |
|  | Longer-term | 2 [6,17] | 102 | 1.79 [-5.77, 9.35] mmHg |  | 0 | |
|  | *Overall subgroup differences* |  |  |  | 0.0002 | 85 | |
| Abbreviations: apo A-I, apolipoprotein A-I; apo B, apolipoprotein B; DBP, diastolic blood pressure; HDL-C, HDL cholesterol; LDL-C, LDL cholesterol; MFGM, milk fat globule membrane; MPL, milk polar lipids; NA, not applicable; SA, sensitivity analysis; SBP, systolic blood pressure; TC, total cholesterol; TG, triacylglycerol; WMD, weighted mean difference.  ¹Data are presented as weighted mean differences (95% CI). Inverse variance random‑effects meta‑analyses were used to pool effect sizes. Subgroup analyses were exploratory and conducted only for outcomes with sufficient data (≥ 5 trials overall and ≥ 2 trials per subgroup level). These analyses were not powered to detect definitive subgroup differences and should be interpreted as hypothesis‑generating. Outcomes without adequate data for subgroup or sensitivity analyses are not included. ^2^Subgroups were defined by: 1) baseline health status (healthy vs. “at-risk” [overweight/obesity or elevated fasting circulating LDL cholesterol]); 2) intervention duration (short-term ≤ 4 wk vs. longer-term > 4 wk); and 3) intervention dosage (low-dose < 300 mg/d vs. high-dose ≥ 300 mg/d). Only subgroup analyses meeting prespecified minimum data requirements (≥ 5 studies overall and ≥ 2 studies per subgroup level) are presented. ^3^Sensitivity analyses assessed baseline imbalance that was defined as a between-group difference > ±10% of clinically desirable upper limits of fasting circulating cholesterol profile and fasting glucose concentrations. Based on these criteria, baseline imbalance was identified for LDL cholesterol and HDL cholesterol in Ohlsson et al. [12] and Calvo et al. [17], and for TG in Ohlsson et al. [12], Severins et al. [7], and Calvo et al. [17]. An additional sensitivity analysis excluding Zajac et al. [18] was conducted due to the inclusion of participants with medically managed type 2 diabetes, allowing assessment of its influence on pooled estimates.  ^4^ Indicates results for between-subgroup differences (interaction effects). Statistical significance was set at P < 0.05. | | | | | | |  |

**Supplemental Table 5A.** Narrative synthesis of fasting blood lipid and lipid-related biomarkers from eligible RCTs of MFGM/MPL^1^

| **Covidence study ID** | **First author, year (country)** | **Health status** | **Study completers, n (%, M/F)** | **Age (mean or range)** | **BMI (mean or range)**  (**kg/m²)** | **Study design, type of dietary intervention, duration of intervention and washout** | **Study arms, n** | **Intervention** | **Comparator** | **Relevant outcomes** | **Results** | **Industrial funding (yes/no)** | **Overall**  **RoB** |
| --- | --- | --- | --- | --- | --- | --- | --- | --- | --- | --- | --- | --- | --- |
| Ohlsson 2009 | Ohlsson et al, 2009 (Sweden) [12] | Healthy | PL-enriched buttermilk: 29 (69/31)    Control: 19 (68/32) | 20-65 y | MPL-enriched buttermilk: 24.1    Control: 24.7 | Parallel,  semi-controlled, intervention: 4-wk, (1-wk run in) | 2 | 350 mL buttermilk drink enriched with Lacprodan PL-20 (1950 mg SL/d)  Supplied by: Arla Foods Amba | Placebo drink made with skim milk powder with PL replaced by 2/3 egg PL isolate + 1/3 butter oil. (238 mg SL/d) | lipoprotein(a) | ↔ lipoprotein(a) vs. control | Yes | Some concerns |
| Conway 2013 | Conway et al., 2013 (Canada) [13] | Healthy  LDL-C < 5 mmol/L  BP < 140/90 mmHg   10 y Framingham risk < 10% | 34 (44/56) | 18-65 y | ≤ 35 | Crossover, placebo-controlled, semi-controlled, intervention: 4-wk, (no washout) | 2 | 45 g/d buttermilk powder mixed with 250 mL water  (187.5 mg MPL/d )  Supplied by: Westland Milk Products, Hokitika, New Zealand | 45 g/d macro/micronutrient-matched dairy-based powder (lacking MFGM), mixed with 250 mL water  (34.6 mg PL/d) | Apo B48, Apo B100 | ↔ Apo B48, Apo B100 vs. control | Yes | Some concerns |
| Ramprasath 2013 | Ramprasath et al., 2013 (Canada) [14] | Healthy    Plasma TC 5.2 mmol/L;    LDL-C: 3.1 mmol/L | 10 (50/50) | 18-45 y | 23 ± 2 | Crossover, fully controlled, intervention: 2-wk (4-wk washout) | 2 | Diet with 1 g milk SM, dissolved in corn oil and added to the diet (1000 mg SM/d)  SM supplied by: Avanti Polar Lipids, Inc. Alabaster, Alabama, USA | Identical diet without SM. | VLDL-C | ↔ VLDL-C vs. control | No | Some concerns |
| Severins 2014 | Severins et al, 2014 (Netherlands) [7] | Mildly hypercholesterolemic    Elevated serum TC: 5.3-8.6 mmol/L | Buttermilk:  23 (57/43)    Skimmed milk: 25 (44/56) | 18-70 y | 23-34 | Parallel, placebo-controlled, semi-controlled, intervention: 12-wk (2-wk run-in) | 4    Extracted: 2 | 80 mL/d traditional buttermilk (0.09% MPL)   Supplied by: Coppelmans BV, Waalre, Nerherlands | 80 mL/d skimmed milk (0.01% PL) | Apo B100 | ↔ Apo B100 vs. control | Yes | Low risk |
| Rosqvist 2015 | Rosqvist et al, 2015 (Sweden) [16] | OW/OB,   Elevated plasma LDL-C: 2.0-4.5 mmol/L | MFGM diet: 26 (31/69)    Control diet: 20 (20/80) | 20-70 y | 25-37 | Parallel, semi-controlled, intervention: 8-wk | 2 | 1 dL/d whipping cream (rich in intact MFGM, 19.8 mg MPL/d) + 1 dL/d fat-free milk + 1 scone    Whipping cream supplied by: Skånemejerier AB, Sweden | 1 dL/d fat-free milk (1.3 mg PL/d) + 1 scone/d | LDL:HDL, VLDL-C, non-HDL-C | ↔ LDL:HDL ratio, VLDL-C vs. control; ↓ non-HDL-C in MFGM vs. ↑ in control | Yes | Some concerns |
| Hari 2015 | Hari et al, 2015 (Japan) [15] | Healthy | MFGM: 16 (50/50)    Placebo: 16 (50/50) | 20-64 y | MFGM: 23.1 ± 1.7     Placebo: 23.0 ± 3.3 | Parallel, placebo-controlled, semi-controlled, intervention: 4-wk | 2 | 6.5 g MFGM/d (231 mg SM/d) in the form of a tablet  Supplied by: NR | 6.5 g/d whole milk powder in the form of a tablet (4 mg SM/d) | NEFA | ↔ NEFA vs. control | NR | Low risk |
| Vors 2020 | Vors et al, 2020 (France) [11]  VALOBAB-C study | OW/OB,    Abdominal obesity: WC: > 80 cm;    serum TC < 11 mmol/L; serum HDL-C < 1.6 mmol/L; serum TG < 3 mmol/L | 3 g MPL: 19 (0/100)    5 g MPL: 20 (0/100)    Control: 19 (0/100) | ≤ 75 y | 25-35 | Parallel, semi-controlled, intervention: 4 wk, (1-wk run-in) | 3 | 100 g/d cream cheese enriched with buttermilk concentrate-derived 3 g or 5 g MPL/d   Buttermilk concentrate supplied by: Candia, Quimper, France | 100 g/d full fat cream cheese, devoid of MPL | Apo B48, Apo B48:Apo B ratio, gut microbiota, SCFA, coprostanol, coprostanol:cholesterol | ↓ Apo B48, Apo B48:Apo B ratio (5 g vs control and 3 g MPL/d); | Yes | Some concerns |
| Abbreviations: Apo B100, apolipoprotein B100; Apo B48, apolipoprotein B48; F, females; HDL-C, HDL cholesterol; LDL-C, LDL cholesterol; M, males; MFGM, milk fat globule membrane; MPL, milk polar lipids; NEFA, non-esterified fatty acids; Non-HDL-C, non-HDL cholesterol; NR, not reported; OB, obese; OW, overweight; PL, phospholipids; RoB, risk of bias; SL, sphingolipid; SM, sphingomyelin; TC, total cholesterol; TG, triacylglycerol; VLDL-C, very-low-density lipoprotein cholesterol; WC, waist circumference. ^1^Fully controlled intervention: all foods and beverages were provided to participants for the duration of the intervention, either for home consumption or consumed on-site (e.g., metabolic ward or research facility). Semi-controlled intervention: experimental foods or supplements were provided to participants, while the remainder of the habitual diet was self-selected. | | | | | | | | | | | | | |

**Supplemental Table 5B.** Narrative synthesis of fasting inflammatory markers from eligible RCTs of MFGM/MPL^1^

| **Covidence study ID** | **First author, year (country)** | **Health status** | **Study completers, n (%, M/F)** | **Age (mean or range)** | **BMI (mean or range)**  **(kg/m²)** | **Study design, type of dietary intervention, duration of intervention and washout** | **Study arms, n** | **Intervention** | **Comparator** | **Relevant outcomes** | **Results** | **Industrial funding (yes/no)** | **Overall**  **RoB** |  |
| --- | --- | --- | --- | --- | --- | --- | --- | --- | --- | --- | --- | --- | --- | --- |
| Weiland 2016 | Weiland et al, 2016 (Germany) [6]  Trial 1 | OW/OB,      fasting glucose < 7.0 mmol/L | 62 (100/0) | 50-76 y | ≥ 27 | Parallel, placebo- controlled, semi-controlled, intervention: 8-wk | 2 | 200 ml/day semi-skimmed milk (1.5% fat) + 2 g MPL (Lipamin M20 powder, 5.4% of drink) (2 g MPL/d)  Lipamine M20 supplied by: Lecico, GmbH | 200 ml/d semi-skimmed milk enriched with cream and skimmed milk concentrate providing 2 g milk fat | IL-6, sICAM | ↔ IL-6, sICAM vs. control | No | Low risk |  |
| Zajac 2025 | Zajac et al, 2025 (Australia) [18] | Generally healthy^2^    subjective memory complaints (MAC-Q ≥ 25)    free from MCI/dementia (MoCA > 22) | Completers:  Control: 76 Low-dose: 75  High-dose: 71    sex breakdown NR for completers | 55-76 y | 18.5 - 35 | Parallel, placebo-controlled, semi-controlled, intervention: 16-wk | 3 | Skim-milk powders fortified with MFGM Phospholipid 100; 50 g/d (two 25 g servings mixed with water)  Low-dose MFGM: 1.7 g MPL/d (per 50 g powder)   High-dose MFGM: 4.0 g MPL/d (per 50 g powder)   (50 g powder was mixed with 200 mL water)  Supplied by: Fonterra Dairy Co-operative, New Zealand | 50 g/d rice starch placebo powder, energy-matched and visually identical, containing 10% whole milk powder  (50 g powder was mixed with 200 mL water) | TNF-α | ↔ (intention-to-treat) TNF-α, ↓ (per-protocol) TNF-α vs. control | Yes | Some concerns |  |
| Abbreviations: F, females; M, males; MAC-Q, memory complaints questionnaire; MCI, mild cognitive impairment; MFGM, milk fat globule membrane; MoCA, Montreal cognitive assessment; MPL, milk polar lipids; NR, not reported; OB, obese; OW, overweight; RoB, risk of bias; sICAM, soluble intercellular adhesion molecule; TNF-α, tumor necrosis factor-alpha. ^1^Semi-controlled intervention: experimental foods or supplements were provided to participants, while the remainder of the habitual diet was self-selected.  ^2^Baseline HbA1c values across study arms were within the normoglycemic range (< 5.7%). | | | | | | | | | | | | | | |

**Supplemental Table 5C.** Narrative synthesis of gut microbiota composition and fecal metabolic outcomes from eligible RCTs of MFGM/MPL^1^

| **Covidence study ID** | **First author, year (country)** | **Health status** | **Study completers, n (%, M/F)** | **Age (mean or range)** | **BMI (mean or range)**  **(kg/m²)** | **Study design, type of dietary intervention, duration of intervention and washout** | **Study arms, n** | **Intervention** | **Comparator** | **Relevant outcomes** | **Results** | **Industrial funding (yes/no)** | **Overall RoB** |
| --- | --- | --- | --- | --- | --- | --- | --- | --- | --- | --- | --- | --- | --- |
| Vors 2020 | Vors et al, 2020 (France) [11]  VALOBAB-C study | OW/OB,    Abdominal obesity: WC: >80 cm;    serum TC < 11 mmol/L; serum HDL-C < 1.6 mmol/L; serum TG < 3 mmol/L | 3 g MPL: 19 (0/100)    5 g MPL: 20 (0/100)    Control: 19 (0/100) | ≤ 75 y | 25-35 | Parallel, semi-controlled, intervention: 4 wk, (1-wk run-in) | 3 | 100 g/d cream cheese enriched with buttermilk concentrate-derived 3 g or 5 g MPL/d   Buttermilk concentrate supplied by: Candia, Quimper, France | 100 g/d full fat cream cheese, devoid of MPL | Gut microbiota, SCFA, coprostanol, coprostanol:cholesterol | ↔ major fecal microbiota groups; fecal SCFA; ↑ coprostanol; ↑ coprostanol:cholesterol ratio vs control | Yes | Some concerns |
| Abbreviations: F, females; HDL-C, HDL cholesterol; M, males; MFGM, milk fat globule membrane; MPL, milk polar lipids; OB, obese; OW, overweight; RoB, risk of bias; SCFA, short chain fatty acids; TC, total cholesterol; TG, triacylglycerol; WC, waist circumference. ^1^Semi-controlled intervention: experimental foods or supplements were provided to participants, while the remainder of the habitual diet was self-selected. | | | | | | | | | | | | | |

**Supplemental Table 5D.** Narrative synthesis of cognitive outcomes from eligible RCTs of MFGM/MPL^1^

| **Covidence study ID** | **First author, year (country)** | **Health status** | **Study completers, n (%, M/F)** | **Age (mean or range)** | **BMI (mean or range) (kg/m²)** | **Study design, type of dietary intervention, duration of intervention and washout** | **Study arms, n** | **Intervention** | **Comparator** | **Relevant outcomes** | **Results** | **Industrial funding (yes/no)** | **Overall**  **RoB** |
| --- | --- | --- | --- | --- | --- | --- | --- | --- | --- | --- | --- | --- | --- |
| Schubert 2011 | Schubert et al, 2011 (Germany) [20] | Healthy,  chronically stressed | 75 (100/0) | 30-51 y | 1% MPL drink: 26.46   0.5% MPL drink: 28.53   Control drink: 26.90 | Parallel, placebo-controlled, semi-controlled, intervention: 12-wk | 3 | 250 mL/d bovine milk drink enriched with 0.5% and 1% MPL (Lacprodan PL-20),  (150 mg and 300 mg MPL/d respectively)  Lacprodan PL-20 supplied by: Arla Foods Ingredients, Viby, Denmark | 250 mL/d placebo bovine milk drink | Memory performance | ↔ Overall memory; ↑ visual memory after stress (high‑dose MPL group)  s) vs. control | Yes | Some concerns |
| Boyle 2019 | Boyle et al, 2019 (United Kingdom) [21] | Healthy | Cognitive tests: PL: 26 (100/0)   Placebo: 26 (100/0) | 21.4 y | < 30 | Parallel, semi-controlled, intervention: 6 wk | 2 | 250 ml/d milk-based drink enriched with MPL (2.7 g MPL/d)  Supplied by: Arla Foods Ingredients, Viby, Denmark | 250 ml/d milk-based drink no MPLs | Working memory performance, executive control function. | ↔ Overall performance; ↓ repeat-trial cost (faster reaction time) with MPL vs. control | Yes | Low risk |
| Calvo 2023 | Calvo et al, 2023 (Spain) [17] | Healthy or mildly cognitively impaired | MFGM: 19 (42/58)    Control: 21 (48/52) | > 60 y | MFGM: 25.7-29.3     Control: 25.4-29.6 | Parallel, placebo-controlled, semi-controlled, intervention: 14-wk | 2 | 200 mL/d MFGM-enriched milk drink (~0.38 g MPL/d)   Supplements produced in collaboration with: INNOLACT S.L., Lugo, Spain and the Aula de Productos L´acteos, APLTA, USC, Spain | 200 mL/d skim milk (~0.001 g PL/d) | Cognitive function | ↔ Global cognition; ↑ short-delay cued recall (in females only) vs. control | No | Some concerns |
| Zajac 2025 | Zajac et al, 2025 (Australia) [18] | Generally healthy^2^    subjective memory complaints (MAC-Q ≥ 25)    free from MCI/dementia (MoCA > 22) | Completers:  Control: 76 Low-dose: 75  High-dose: 71    sex breakdown NR for completers | 55-76 y | 18.5 - 35 | Parallel, placebo-controlled, semi-controlled, intervention: 16-wk | 3 | Skim-milk powders fortified with MFGM Phospholipid 100; 50 g/d (two 25 g servings mixed with water)  Low-dose MFGM: 1.7 g MPL/d (per 50 g powder)   High-dose MFGM: 4.0 g MPL/d (per 50 g powder)   (50 g powder was mixed with 200 mL water)  Supplied by: Fonterra Dairy Co-operative, New Zealand | 50 g/d rice starch placebo powder, energy-matched and visually identical, containing 10% whole milk powder  (50 g powder was mixed with 200 mL water) | Cognitive function assessed with COMPASS, RBANS | ↔ RBANS; ↔ COMPASS scores vs. control | Yes | Some concerns |
| Abbreviations: COMPASS, Computerised Mental Performance Assessment System; F, females; M, males; MAC-Q, memory complaints questionnaire; MCI, mild cognitive impairment; MoCA, Montreal Cognitive Assessment; MFGM, milk fat globule membrane; MPL, milk polar lipids; NR, not reported; PL, phospholipids; RBANS, Repeatable Battery for the Assessment of Neuropsychological Status; RoB, risk of bias; SL, sphingolipid; SM, sphingomyelin. ^1^Semi-controlled intervention: experimental foods or supplements were provided to participants, while the remainder of the habitual diet was self-selected.  ^2^Baseline HbA1c values across study arms were within the normoglycemic range (< 5.7%). | | | | | | | | | | | | | |

**Supplemental Table 6.** Synthesis of characteristics of eligible RCTs on the impact of milk fat globule membrane and its associated polar lipids on cardiometabolic disease risk markers in the postprandial state

| **Covidence study ID** | **First author, year (country)** | **Health status** | **Study completers, n (%, M/F)** | **Age (mean or range)** | **BMI (mean or range) (kg/m²)** | **Study design, duration of washout (d), duration of postprandial period (h)** | **Study arms (n), standardized meal components** | **Intervention** | **Comparator** | **Relevant outcomes** | **Results** | **Industrial funding (yes/no)** | **Overall RoB** |
| --- | --- | --- | --- | --- | --- | --- | --- | --- | --- | --- | --- | --- | --- |
| Ohlsson 2010 | Ohlsson et al., 2010 (Sweden) [22] | Healthy | 18 (100/0) | 22-65 y | 24.3 | Crossover,  10-14 d  washout  period, 7 h | 2, Standardized meal consumed with test drinks:  Semolina pudding, cream, butter, bread, liver paste, blackberry jam; ~2683 kJ and ~40 g fat | 350 mL buttermilk drink enriched with MPLs providing 975 mg/serving SL (700 mg SM) (Lacprodan PL-20).  Supplied by: Arla Foods Amba, Sweden | 350 mL skim milk powder–based drink with native PLs replaced by 2/3 egg PL isolate (Fresenius Biotech, Munich) and 1/3 butter oil (119 mg/serving SL (SM content NR). | TG, TC, LDL-C, HDL-C, cholesterol in TG-rich lipoproteins, apo A-I, apo B, apo B:apo A-I ratio, glucose, insulin, PO: NR | ↔ iAUC₀₋₇ or 7 h concentrations for TG, TC, LDL-C, HDL-C, cholesterol in TG-rich lipoproteins, apo A-I, apo B, apo B:apo A-I ratio, glucose and insulin between MPL and control drink | Yes | Some concerns |
| Abbreviations: apo A-I, apolipoprotein A-I; apo B, apolipoprotein B; F, females; HDL-C, HDL cholesterol; LDL-C, LDL cholesterol; M, males; MPL, milk polar lipid; NR, not reported; PO, primary outcome; RoB, risk of bias; SL, sphingolipid; SM, sphingomyelin; TG, triacylglycerol; TC, total cholesterol. | | | | | | | | | | | | | |

**Supplemental Table 7.** GRADE evidence profiles the effects of milk fat globule membrane (MFGM) and its associated milk polar lipids (MPLs) on critical fasting cardiometabolic disease risk markers in disease-free adults

**Question:** Does consumption of MFGM and its associated MPLs improve fasting cardiometabolic disease risk markers, compared to isoenergetic alternative dairy products or supplements with low MFGM/MPL content?

| **Certainty assessment** | | |  |  |  |  |  |  |  |  |  |
| --- | --- | --- | --- | --- | --- | --- | --- | --- | --- | --- | --- |
| **Total studies (references)** | **Study design** | **Risk of bias** | **Inconsistency^7^** | **Indirectness^8^** | **Imprecision** | **Other considerations^11^** | **Outcome** | **No of participants (studies)** | **Effect (Absolute [95% CI])** | **Certainty** | **Importance of outcome^12^** |
| Blood lipids | | | | | | | | | | | |
| 10 [6,7,11–18] | RCT | Serious^1^ | Not serious | Not serious | Not serious^9^ | None | TC | 600 (10 RCTs) | WMD:  -0.18 [0.30, -0.07] mmol/L | ⨁⨁⨁◯ Moderate | Critical |
| 10 [6,7,11–18] | RCT | Serious^1^ | Not serious | Not serious | Not serious^9^ | None | LDL-C | 600 (10 RCTs) | WMD:  -0.12 [0.22, -0.03] mmol/L | ⨁⨁⨁◯ Moderate | Critical |
| 10 [6,7,11–18] | RCT | Serious^1^ | Not serious | Not serious | Serious^10^ | None | HDL-C | 600 (10 RCTs) | WMD:   -0.01 [-0.06, 0.03] mmol/L | ⨁⨁◯◯  Low | Critical |
| 6 [6,7,11,13,14,16] | RCT | Serious^2^ | Not serious | Not serious | Not serious^9^ | None | TC:HDL-C ratio | 258 (6 RCTs) | WMD:   -0.17 [-0.33, -0.01] | ⨁⨁⨁◯ Moderate | Critical |
| 5 [6,11,12,16,17] | RCT | Serious^3^ | Not serious | Not serious | Not serious^9^ | None | Apo B | 254 (5 RCTs) | WMD:   -0.05 [-0.09, -0.01] g/L | ⨁⨁⨁◯ Moderate | Critical |
| 10 [6,7,11–18] | RCT | Serious^1^ | Not serious | Not serious | Serious^10^ | None | TG | 600 (10 RCTs) | WMD:   -0.09 [-0.20, 0.03] mmol/L | ⨁⨁◯◯  Low | Critical |
| Glycemic control | | | | | | | | | | | |
| 5 [6,11,15–17] | RCT | Serious^4^ | Not serious | Not serious | Serious^10^ | None | Glucose | 238 (5 RCTs) | WMD:  -0.03 [-0.18, 0.12] mmol/L | ⨁⨁◯◯  Low | Critical |
| 3 [6,11,16] | RCT | Serious^5^ | Not serious | Not serious | Serious^10^ | None | Insulin | 166 (3 RCTs) | WMD:  -0.80 [-2.12, 0.52] mIU/L | ⨁⨁◯◯  Low | Critical |
| 3 [6,11,16] | RCT | Serious^5^ | Not serious | Not serious | Serious^10^ | None | HOMA-IR ^13^ | 166 (3 RCTs) | WMD:   -0.27 [-0.53, -0.00] | ⨁⨁◯◯  Low | Critical |
| Blood pressure | | | | | | | | | | | |
| 5 [6,11,13,15,17] | RCT | Serious^6^ | Not serious | Not serious | Serious^10^ | None | Systolic | 226 (5 RCTs) | WMD:   -0.02 [-4.73, 4.69] mmHg | ⨁⨁◯◯  Low | Critical |
| 5 [6,11,13,15,17] | RCT | Serious^6^ | Not serious | Not serious | Serious^10^ | None | Diastolic | 226 (5 RCTs) | WMD:   -1.05 [-3.78, 1.68] mmHg | ⨁⨁◯◯  Low | Critical |
| Abbreviations: 95% CI, 95% confidence interval; apo, apolipoprotein; GRADE, Grading of Recommendations, Assessment, Development and Evaluation; HDL-C, HDL cholesterol; HOMA-IR, homeostasis model assessment of insulin resistance; LDL-C, LDL cholesterol; WMD, weighted mean difference; RCT, randomized controlled trial; TG, triacylglycerol; TC, total cholesterol.  ^1^Risk of bias was rated low in 3 studies were [6,7,15] and some concerns in 7 studies [11–14,16–18]. The domain was downgraded by one level due to the proportion of studies with some concerns and their contribution to the pooled effect estimate.  ^2^Risk of bias was low in 2 studies [6,7] and some concerns in 4 studies [11,13,14,16]. The domain was downgraded by one level due to the proportion of studies with some concerns and their contribution to the pooled effect estimate.  ^3^Risk of bias was low in 1 study [6] and some concerns in 4 studies [11,12,16,17]. The domain was downgraded by one level due to the proportion of studies with some concerns and their contribution to the pooled effect estimate.  ^4^Risk of bias was low in 2 studies [6,15] and some concerns in 3 studies [11,16,17]. The domain was downgraded by one level due to the proportion of studies with some concerns and their contribution to the pooled effect estimate.  ^5^Risk of bias was low in 1 study [6] and some concerns in 2 studies [11,16]. The domain was downgraded by one level due to the proportion of studies with some concerns and their contribution to the pooled effect estimate.  ^6^Risk of bias was low in 2 studies [6,15] and some concerns in 3 studies [11,13,17]. The domain was downgraded by one level due to the proportion of studies with some concerns and their contribution to the pooled effect estimate.  ^7^Not downgraded for inconsistency. Despite variation in interventions and outcomes, effect direction and magnitude were sufficiently consistent to justify a single pooled estimate. Heterogeneity was low (*I²* < 50%), supporting the decision not to downgrade.  ^8^Not downgraded for indirectness as study populations, exposures and comparators were relevant to review question. One included study [18] which recruited generally healthy adults, did not report individual‑level diabetes status, glucose‑lowering medication use (as permitted by the eligibility criteria), or analyses restricted to non‑diabetic participants; however, baseline HbA1c values were within the normoglycemic range with low variability, and sensitivity analyses excluding this study did not materially alter pooled effect estimates. Indirectness was therefore judged as not serious.  ^9^Not downgraded as no evidence of imprecision.  ^10^The 95% confidence interval crossed the threshold of important benefit or harm, including the possibility of no effect. Therefore, the domain was downgraded by one level for imprecision.  ^11^There was no suggestion of publication bias based on study characteristics and the reasons for exclusion during screening.  ^12^The GRADE approach categorises outcomes by importance. Outcomes rated as ‘critical’ are considered most important for informing public health guidelines and policy [23].  ^13^HOMA‑IR is a derived measure calculated from fasting glucose and insulin concentrations. As these component biomarkers were rated ‘low certainty’, the certainty for HOMA‑IR was aligned accordingly in line with GRADE guidance for composite or calculated outcomes. | | | | | | | | | | | |

| (A) | (B) |
| --- | --- |

**Supplemental Figure 1**. Study level of bias assessment of randomized controlled trials reporting the impact of milk fat globule membrane and its associated polar lipids on cardiometabolic disease and cognitive function risk markers in the (A) fasted and (B) postprandial state using the Cochrane Risk of Bias tool 2.0.

| (A) | (B) |
| --- | --- |

| (C) | (D) |
| --- | --- |

**Supplemental Figure 2**. Funnel plots for random‑effects meta‑analyses including ≥ 10 studies, assessing the effect of MFGM/MPL intake versus control on fasting circulating (A) total cholesterol, (B) LDL cholesterol, (C) HDL cholesterol, and (D) triacylglycerol. Funnel plots were generated to explore potential small‑study effects and publication bias. Egger’s regression test was used to evaluate funnel plot asymmetry.

**Supplemental Figure 3**. Forest plot of subgroup analyses of MFGM/MPL versus control on fasting circulating total cholesterol (n = 600 participants). Subgroups were defined as follows: (1) baseline health status (healthy vs. at‑risk, where “at‑risk” included individuals with overweight/obesity or elevated fasting circulating LDL cholesterol); (2) intervention duration (short‑term ≤ 4 weeks vs. longer‑term > 4 weeks); and (3) intervention dosage (low‑dose < 300 mg/d vs. high‑dose ≥ 300 mg/d). Effect sizes were calculated as weighted mean differences (95% CIs) using an inverse variance random-effects model. The restricted maximum likelihood method was employed to estimate heterogeneity variance. The Hartung-Knapp-Sidik-Jonkman correction was applied to estimate the 95% CIs of the summary effects. Horizontal lines represent 95% CIs for individual study estimates; green boxes represent individual study effect estimates, with box size proportional to study weight; and black rhombuses represent the pooled effect estimate, with rhombus width indicating the 95% CI. TC, total cholesterol.

**Supplemental Figure 4**. Forest plot of subgroup analyses of MFGM/MPL versus control on fasting circulating LDL cholesterol (n = 600 participants). Subgroups were defined as follows: (1) baseline health status (healthy vs. at‑risk, where “at‑risk” included individuals with overweight/obesity or elevated fasting circulating LDL cholesterol); (2) intervention duration (short‑term ≤ 4 weeks vs. longer‑term > 4 weeks); and (3) intervention dosage (low‑dose < 300 mg/d vs. high‑dose ≥ 300 mg/d). Effect sizes were calculated as weighted mean differences (95% CIs) using an inverse variance random-effects model. The restricted maximum likelihood method was employed to estimate heterogeneity variance. The Hartung-Knapp-Sidik-Jonkman correction was applied to estimate the 95% CIs of the summary effects. Horizontal lines represent 95% CIs for individual study estimates; green boxes represent individual study effect estimates, with box size proportional to study weight; and black rhombuses represent the pooled effect estimate, with rhombus width indicating the 95% CI. LDL-C, LDL cholesterol.

**Supplemental Figure 5**. Forest plot of subgroup analyses of MFGM/MPL versus control on fasting circulating HDL cholesterol (n = 600 participants). Subgroups were defined as follows: (1) baseline health status (healthy vs. at‑risk, where “at‑risk” included individuals with overweight/obesity or elevated fasting circulating LDL cholesterol); (2) intervention duration (short‑term ≤ 4 weeks vs. longer‑term > 4 weeks); and (3) intervention dosage (low‑dose < 300 mg/d vs. high‑dose ≥ 300 mg/d). Effect sizes were calculated as weighted mean differences (95% CIs) using an inverse variance random-effects model. The restricted maximum likelihood method was employed to estimate heterogeneity variance. The Hartung-Knapp-Sidik-Jonkman correction was applied to estimate the 95% CIs of the summary effects. Horizontal lines represent 95% CIs for individual study estimates; green boxes represent individual study effect estimates, with box size proportional to study weight; and black rhombuses represent the pooled effect estimate, with rhombus width indicating the 95% CI. HDL-C, HDL cholesterol

**Supplemental Figure 6**. Forest plot of subgroup analyses of MFGM/MPL versus control on fasting circulating TC:HDL cholesterol ratio (n = 600 participants). Subgroups were defined as follows: (1) baseline health status (healthy vs. at‑risk, where “at‑risk” included individuals with overweight/obesity or elevated fasting circulating LDL cholesterol); (2) intervention duration (short‑term ≤ 4 weeks vs. longer‑term > 4 weeks); and (3) intervention dosage (low‑dose < 300 mg/d vs. high‑dose ≥ 300 mg/d). Effect sizes were calculated as weighted mean differences (95% CIs) using an inverse variance random-effects model. The restricted maximum likelihood method was employed to estimate heterogeneity variance. The Hartung-Knapp-Sidik-Jonkman correction was applied to estimate the 95% CIs of the summary effects. Horizontal lines represent 95% CIs for individual study estimates; green boxes represent individual study effect estimates, with box size proportional to study weight; and black rhombuses represent the pooled effect estimate, with rhombus width indicating the 95% CI. TC, total cholesterol; HDL-C, HDL cholesterol.

**Supplemental Figure 7**. Forest plot of subgroup analyses of MFGM/MPL versus control on fasting circulating apolipoprotein B (n = 600 participants). Subgroups were defined as follows: (1) baseline health status (healthy vs. at‑risk, where “at‑risk” included individuals with overweight/obesity or elevated fasting circulating LDL cholesterol) and (2) intervention duration (short‑term ≤ 4 weeks vs. longer‑term > 4 weeks). Effect sizes were calculated as weighted mean differences (95% CIs) using an inverse variance random-effects model. The restricted maximum likelihood method was employed to estimate heterogeneity variance. The Hartung-Knapp-Sidik-Jonkman correction was applied to estimate the 95% CIs of the summary effects.  Horizontal lines represent 95% CIs for individual study estimates; green boxes represent individual study effect estimates, with box size proportional to study weight; and black rhombuses represent the pooled effect estimate, with rhombus width indicating the 95% CI. Apo B, apolipoprotein B.

**Supplemental Figure 8**. Forest plot of subgroup analyses of MFGM/MPL versus control on fasting circulating triacylglycerol (n = 600 participants). Subgroups were defined as follows: (1) baseline health status (healthy vs. at‑risk, where “at‑risk” included individuals with overweight/obesity or elevated fasting circulating LDL cholesterol); (2) intervention duration (short‑term ≤ 4 weeks vs. longer‑term > 4 weeks); and (3) intervention dosage (low‑dose < 300 mg/d vs. high‑dose ≥ 300 mg/d). Effect sizes were calculated as weighted mean differences (95% CIs) using an inverse variance random-effects model. The restricted maximum likelihood method was employed to estimate heterogeneity variance. The Hartung-Knapp-Sidik-Jonkman correction was applied to estimate the 95% CIs of the summary effects. Horizontal lines represent 95% CIs for individual study estimates; green boxes represent individual study effect estimates, with box size proportional to study weight; and black rhombuses represent the pooled effect estimate, with rhombus width indicating the 95% CI.  TG, triacylglycerol

**Supplemental references**

1. Sterne JAC, Savović J, Page MJ, Elbers RG, Blencowe NS, Boutron I, Cates CJ, Cheng HY, Corbett MS, Eldridge SM, et al., RoB 2: a revised tool for assessing risk of bias in randomised trials, BMJ; (366) (2019). doi: 10.1136/bmj.l4898.
2. Li F, He R, Yue Z, Yi H, Lu L, Zhang L, Shi J, Zheng C, Jiao J, Peng J, et al., Effect of a 12-mo intervention with whey protein powder on cognitive function in older adults with mild cognitive impairment: a randomized controlled trial, Am. J. Clin. Nutr.; (121) (2024) 256-264. doi: 10.1016/j.ajcnut.2024.11.019.
3. Daly RM, Gianoudis J, de Ross B, O’Connell SL, Kruger M, Schollum L, Gunn C, Effects of a multinutrient-fortified milk drink combined with exercise on functional performance, muscle strength, body composition, inflammation, and oxidative stress in middle-aged women: A 4-month, double-blind, placebo-controlled, randomized trial, Am. J. Clin. Nutr.; (112) (2020) 427–46. doi: 10.1093/ajcn/nqaa126.
4. Soga S, Ota N, Shimotoyodome A, Dietary milk fat globule membrane supplementation combined with regular exercise improves skeletal muscle strength in healthy adults: a randomized double-blind, placebo-controlled, crossover trial, Nutr. J.; (14) (2015) 85. doi: 10.1186/s12937-015-0073-5.
5. Pokala A, Quarles WR, Ortega-Anaya J, Jimenez-Flores R, Cao S, Zeng M, Hodges JK, Bruno RS, Milk-Fat-Globule-Membrane-Enriched Dairy Milk Compared with a Soy-Lecithin-Enriched Beverage Did Not Adversely Affect Endotoxemia or Biomarkers of Gut Barrier Function and Cardiometabolic Risk in Adults with Metabolic Syndrome: A Randomized Controlled Crossover Trial, Nutrients; (15) (2023) 3259. doi: 10.3390/nu15143259.
6. Weiland A, Bub A, Barth SW, Schrezenmeir J, Pfeuffer M, Effects of dietary milk- and soya-phospholipids on lipid-parameters and other risk indicators for cardiovascular diseases in overweight or obese men – Two double-blind, randomised, controlled, clinical trials, J. Nutr. Sci.; (5) (2016). doi: 10.1017/jns.2016.9.
7. Severins N, Mensink RP, Plat J, Effects of lutein-enriched egg yolk in buttermilk or skimmed milk on serum lipids & lipoproteins of mildly hypercholesterolemic subjects, Nutr. Metab. Cardiovasc. Dis.; (25) (2015) 210–7. doi: 10.1016/j.numecd.2014.10.003.
8. Beals E, Kamita SG, Sacchi R, Demmer E, Rivera N, Rogers-Soeder TS, Gertz ER, Van Loan MD, German JB, Hammock BD, et al., Addition of milk fat globule membrane-enriched supplement to a high-fat meal attenuates insulin secretion and induction of soluble epoxide hydrolase gene expression in the postprandial state in overweight and obese subjects, J. Nutr. Sci.; (8) (2019) e16. doi: 10.1017/jns.2019.11.
9. Demmer E, Van Loan MD, Rivera N, Rogers TS, Gertz ER, Bruce German J, Smilowitz JT, Zivkovic AM, Addition of a dairy fraction rich in milk fat globule membrane to a high-saturated fat meal reduces the postprandial insulinaemic and inflammatory response in overweight and obese adults, J. Nutr. Sci.; (5) (2016) e14. doi: 10.1017/jns.2015.42.
10. Rogers TS, Demmer E, Rivera N, Gertz ER, German JB, Smilowitz JT, Zivkovic AM, Van Loan MD, The role of a dairy fraction rich in milk fat globule membrane in the suppression of postprandial inflammatory markers and bone turnover in obese and overweight adults: an exploratory study, Nutr. Metab. (Lond).; (14) (2017) 1–9. doi: 10.1186/s12986-017-0189-z.
11. Vors C, Joumard-Cubizolles L, Lecomte M, Combe E, Ouchchane L, Drai J, Raynal K, Joffre F, Meiller L, Le Barz M, et al., Milk polar lipids reduce lipid cardiovascular risk factors in overweight postmenopausal women: towards a gut sphingomyelin-cholesterol interplay, Gut; (69) (2020) 487–501. doi: 10.1136/gutjnl-2018-318155.
12. Ohlsson L, Burling H, Nilsson Å, Long term effects on human plasma lipoproteins of a formulation enriched in butter milk polar lipid, Lipids Health Dis.; (8) (2009). doi: 10.1186/1476-511X-8-44.
13. Conway V, Couture P, Richard C, Gauthier SF, Pouliot Y, Lamarche B, Impact of buttermilk consumption on plasma lipids and surrogate markers of cholesterol homeostasis in men and women, Nutr. Metab. Cardiovasc Dis.; (23) (2013) 1255–62. doi: 10.1016/j.numecd.2013.03.003.
14. Ramprasath VR, Jones PJH, Buckley DD, Woollett LA, Heubi JE, Effect of dietary sphingomyelin on absorption and fractional synthetic rate of cholesterol and serum lipid profile in humans, Lipids Health Dis.; (12) (2013). doi: 10.1186/1476-511X-12-125.
15. Hari S, Ochiai R, Shioya Y, Katsuragi Y, Safety evaluation of the consumption of high dose milk fat globule membrane in healthy adults: a double-blind, randomized controlled trial with parallel group design, Biosci. Biotechnol. Biochem.; (79) (2015) 1172–7. doi: 10.1080/09168451.2015.1012150.
16. Rosqvist F, Smedman A, Lindmark-Mansson H, Paulsson M, Petrus P, Straniero S, Rudling M, Dahlman I, Risérus U, Potential role of milk fat globule membrane in modulating plasma lipoproteins, gene expression, and cholesterol metabolism in humans: a randomized study, Am. J. Clin. Nutr.; (102) (2015) 20–30. doi: 10.3945/ajcn.115.107045.
17. Calvo MV., Loria Kohen V, Díaz-Mardomingo C, García-Herranz S, Baliyan S, Tomé-Carneiro J, Colmenarejo G, Visioli F, Venero C, Fontecha J, Milk fat globule membrane-enriched milk improves episodic memory: A randomized, parallel, double-blind, placebo-controlled trial in older adults, J. Funct. Foods; (111) (2023) 105849. doi: 10.1016/j.jff.2023.105849.
18. Zajac IT, Kakoschke N, Evas S, Kuhn-Sherlock B, Wood K, King R, Arnoldy L, Dekker J, Gunn C, Fuad M, et al., The effect of milk fat globule membrane phospholipid supplementation on cognitive function in older adults with subjective memory complaints—a randomized, double-blind, placebo-controlled trial, Am. J. Clin. Nutr.; (122) (2025) 1161–73. doi: 10.1016/j.ajcnut.2025.09.005.
19. Conway V, Couture P, Gauthier S, Pouliot Y, Lamarche B, Effect of buttermilk consumption on blood pressure in moderately hypercholesterolemic men and women, Nutrition; (30) (2014) 116–9. doi: 10.1016/j.nut.2013.07.021.
20. Schubert M, Contreras C, Franz N, Hellhammer J, Milk-based phospholipids increase morning cortisol availability and improve memory in chronically stressed men, Nutr. Res.; (31) (2011) 413–20. doi: 10.1016/j.nutres.2011.05.012.
21. Boyle NB, Dye L, Arkbåge K, Thorell L, Frederiksen P, Croden F, Lawton C, Effects of milk-based phospholipids on cognitive performance and subjective responses to psychosocial stress: A randomized, double-blind, placebo-controlled trial in high-perfectionist men, Nutrition; (57) (2019) 183–93. doi: 10.1016/j.nut.2018.05.002.
22. Ohlsson L, Burling H, Duan RD, Nilsson A, Effects of a sphingolipid-enriched dairy formulation on postprandial lipid concentrations, Eur. J. Clin. Nutr.; (64) (2010) 1344–9. doi: 10.1038/ejcn.2010.164
23. Schumemann H, Brożek J, Guyatt G, Oxman A, GRADE Handbook. Grading of Recommendations Assessment, Development and Evaluation, Grade Working Group. - References - Scientific Research Publishing, Grade Working Group; (2013).
